# Mitogenomic Insights Into the Ancient Split and Cryptic Diversity of the *Poecilochirus carabi* Complex (Acari: Mesostigmata)

**DOI:** 10.1002/ece3.74501

**Published:** 2026-10-11

**Authors:** Lang Liang, Pei‐Yue Zhao, Hu‐Die He, Tian‐Ci Yi, Dao‐Chao Jin

**Affiliations:** ^1^ The Provincial Key Laboratory for Plant Pest Management of Mountainous Region, Scientific Observing and Experimental Station of Crop Pest in Guiyang, Institute of Entomology, Ministry of Agricultural and Rural Affairs Guizhou University Guiyang P. R. China; ^2^ Guizhou Civil Aviation Vocational College Anshun P. R. China

**Keywords:** cryptic species, forensic acarology, Parasitoidea, phoresy, species delimitation

## Abstract

Phoretic associations with insects are widespread in the Mesostigmata. These temporary symbiotic associations have been important in driving the evolutionary development of this taxon. The phoretic mite genus *Poecilochirus*, which is considered obligately associated with burying beetles, is of great value in forensic entomology. However, due to the morphological conservatism and the difficulty of obtaining adult specimens, the taxonomy of this genus has long been based on deutonymphal characters, and the true species diversity is far from resolved. Here we used next‐generation sequencing to determine, for the first time, the complete mitochondrial genomes of *P. austroasiaticus*, *P. subterraneus* , and two distinct lineages of *P. carabi* (BJ1 and BJ2). All the sequenced mitogenomes contain 37 genes with a gene order identical to that of the ancestral arthropod arrangement and they all show pronounced AT bias and negative GC skew. All 13 protein‐coding genes are under strong purifying selection (*Ka/Ks* < 1). Sliding window analysis identifies *nad2* and *nad5* as nucleotide diversity hotspots, which could serve as high‐resolution markers for future species delimitation. Phylogenetic reconstruction tentatively recovered *Poecilochirus* as paraphyletic with *Parasitus* nested within it (ML = 44%, BI = 0.982), while Parasitinae and Pergamasinae are not reciprocally monophyletic. The *cox1* genetic distance between *P. carabi* BJ1 and BJ2 reached 0.409, with both mPTP and ASAP analyses of the mitogenomic dataset partitioning them into separate candidate molecular operational taxonomic units. Bayesian divergence time estimation revealed that the two lineages diverged in the Eocene about 52.4 million years ago (Ma), before the split between the morphologically recognized species *P. subterraneus* and *P. davydovae* (39.0 Ma). Our results give strong molecular evidence for cryptic speciation within the *P. carabi* complex and provide a solid genomic foundation for the future systematic and taxonomic research in the Parasitidae.

## Graphical abstract

Mitochondrial genomes of *Poecilochirus* mites reveal a paraphyletic genus and uncover deep cryptic speciation within the *P. carabi* complex. Two divergent lineages (BJ1 and BJ2) show a *cox1* genetic distance of 0.409 and an Eocene divergence time of ~52.4 million years ago, predating the split between two morphologically recognized species. These results challenge current morphology‐based taxonomy and provide genomic markers for species delimitation in Parasitidae.

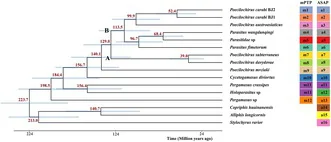

## 1 Introduction

Phoresy is a widespread phenomenon among mesostigmatid mites (Mesostigmata), which are very good at attaching to insects (Bajerlein et al. 2024; Perotti and Braig 2009). Phoresy was previously thought to be only a means of dispersal, but ongoing work has provided numerous lines of evidence suggesting that this behavior can involve a much wider and more profound range of functions (Noriega et al. 2022; Seeman and Walter 2023). For example, the cryptic species *Poecilochirus carabi* and *P. necrophori* have different host preferences for the burying beetles *Nicrophorus vespilloides* and *N. vespillo* , respectively (Nehring et al. 2017; Sun and Kilner 2024). The strong reduction in reproductive fitness when these mites are associated with non‐preferred hosts shows the trade‐offs of the adaptive host specialization. However, this kind of evolutionary pressure is not unidirectional, as hosts may also develop tolerance toward the prevalent phoretic associates. For example, *N. vespilloides* has little fitness cost when coexisting with the typical symbiont of this beetle, *P. carabi*, which hints that there is a subtle and dynamic balance along the mutualism‐antagonism continuum (Nehring et al. 2017).

The phoretic ecology of the genus *Poecilochirus* is well known; its spatial dispersal relies obligately on burying beetle hosts (Silphidae) (Bordas and Perotti 2019; Sun and Kilner 2024). This strong host specificity is very diagnostic in forensic entomology, in particular for the estimation of the post‐mortem interval (PMI) and reconstruction of the micro‐ecological succession on the carcass (Bala and Singh 2019; Ondrejková et al. 2021; Saloña‐Bordas and Perotti 2014). Twenty species have been morphologically described within *Poecilochirus*, but the current taxonomic framework of the genus is based mostly on the morphological characters of the deutonymphs because adults are difficult to obtain in the field. Since the morphological distinctions of the species are very subtle, the real diversity of the genus is not well defined. Indeed, the molecular analysis of Canitz et al. (2022) showed that *P. carabi* comprises at least 17 distinct genetic clusters, and it is very likely that substantial cryptic speciation remains to be taxonomically resolved.

The mitochondrial genome (mitogenome) has become a key molecular marker for animal phylogenetics and systematics (Li et al. 2022; Tang et al. 2019; Wang et al. 2017). Because of the maternal inheritance, linked gene architecture, the mitogenome is important for the reconstruction of the deep evolutionary history and to settle taxonomic ambiguity in the Mesostigmata (Arribas et al. 2020; He et al. 2023; Yang et al. 2023a). The extensive mitochondrial gene order rearrangements reported in the mesostigmatid lineages provide strong phylogenetic signals that can help to clarify the controversial relationships (Li et al. 2019; Liang et al. 2024; Yuan et al. 2024). The combined mitogenomic and nuclear phylogenetic analyses consistently placed the *Varroa* mites in the family Laelapidae (Oh et al. 2024). As for the recent mitogenomic investigation of the genus *Poecilochirus*, it also showed a very close phylogenetic affinity with *Parasitus* (Zhao et al. 2024). All the above demonstrates that mitogenomic data are useful for delineating evolutionary boundaries among closely related mesostigmatid lineages.

Although it has been shown that the mitogenomic data are very useful to solve the mesostigmatid systematics, the low number of available sequences for Poecilochirus did not allow a full understanding of its phylogeny and taxonomy. In this paper, we sequenced the complete mitogenomes of three *Poecilochirus* species (four mitogenomes in total) using next‐generation sequencing (NGS): *P. austroasiaticus* Vitzthum, 1930, *P. subterraneus* (Müller, 1860), and two distinct lineages of *P. carabi* G. Canestrini and R. Canestrini, 1882 (BJ1 and BJ2). We characterized their mitogenomic architecture, in particular the genomic composition and the codon usage bias, systematically. Molecular phylogenetic analyses were then used to assess their taxonomic status and their evolutionary relationships. Using these new sequences together with publicly available mitogenomes of other Parasitidae mites (from GenBank), we were able to construct a robust phylogeny and estimate divergence times across the main lineages. We hope that this study will provide some genomic resources and a good reference for future phylogenetic resolution, for cryptic species delimitation, and for integrative taxonomic revision in the genus *Poecilochirus*.

## 2 Materials and Methods

### 2.1 Sampling and Identification

From Mount Wuling, Miyun District, Beijing, China (117°22′23.31″E, 40°34′50.10″N; 994.32 m a.s.l.), specimens of three *Poecilochirus* species were collected on July 9, 2023. All specimens were phoretically associated with host beetles of the *Nicrophorus* sp. (Nicrophorinae). The two *P. carabi* lineages (BJ1 and BJ2) originated from the same beetle host. The collected specimens were preserved in absolute ethanol and stored at −20°C for molecular work. Five individuals were cleared and permanently mounted in Hoyer's medium for morphological examination under a compound light microscope. Species identification was according to the diagnostic descriptions and taxonomical keys of Hyatt (1980) and Ma (2005). Voucher specimens are deposited in the Institute of Entomology, Guizhou University, Guiyang, China.

### 2.2 DNA Extraction and Sequencing

Genomic DNA for each species was extracted from another five females from the same host beetle using the QIAamp DNA Micro Kit (Qiagen, Hilden, Germany). The DNA integrity was evaluated by 1% agarose gel electrophoresis. The concentration of DNA was determined with a Qubit 4.0 Fluorometer (Thermo Fisher Scientific, Waltham, MA, USA). For mitogenome sequencing of the three *Poecilochirus* species, we sequenced on an Illumina NovaSeq 6000 platform (Berry Genomics, Beijing, China) with a paired‐end 150 bp (PE150) strategy. Each species yielded at least 6 Gb of high‐quality clean reads after stringent quality control and filtering.

### 2.3 Assembly and Annotation

The mitogenomes were assembled with IDBA‐UD (Peng et al. 2012) with parameters as Liang et al. (2023). Due to substantial lineage divergence, the assembly successfully resolved two distinct and complete circular mitogenomes (lineages BJ1 and BJ2) from the pooled sequencing data, with no chimeric breakpoints detected. The biological identities of both lineages were independently validated by NCBI BLAST queries of *cox1* (BJ1 matched *P. carabi* s.l. clade CH‐N.jap with 99.26% identity; BJ2 matched *P. carabi* s.l. clade Eurasia with 98.53% identity). Assembly reliability and the absence of NUMTs were confirmed in Geneious v10.1.3 by high, continuous read coverage (> 100×) and intact open reading frames across all 13 protein‐coding genes (PCGs) without premature stops or frameshifts. Genes were annotated independently using MitoZ (Meng et al. 2019) and MITOS2 (Donath et al. 2019), followed by cross‐checking and manual curation in Geneious v10.1.3. The 22 transfer RNA (tRNA) genes and their secondary structures were predicted by tRNAscan‐SE v2.0 (Lowe and Chan 2016) and ARWEN v1.2.3 (Laslett and Canbäck 2008). The *16S* and *12S* ribosomal RNA (rRNA) genes were initially identified based on their conserved positions relative to flanking tRNA genes, and their identities were subsequently confirmed through sequence alignment with published *Poecilochirus* mitogenomes (Zhao et al. 2024).

### 2.4 Comparative Mitogenomics

The base composition and RSCU of PCGs were calculated by MEGA‐X (Kumar et al. 2018). The compositional skew was calculated using the formulas AT‐skew = (A − T)/(A + T) and GC‐skew = (G − C)/(G + C) (Perna and Kocher 1995). Nucleotide diversity (π) was estimated by a sliding window analysis in DnaSP v6 (Rozas et al. 2017) of 200 bp with a step of 20 bp; *π* was plotted with the midpoint position of each window. A hierarchical clustering analysis based on Euclidean distances of RSCU profiles was performed to study the lineage‐specific codon usage. To assess selective pressures on mitochondrial PCGs, pairwise *Ka/Ks* ratios were calculated using KaKs_Calculator v3.0 (Zhang 2022) under the modified Yang‐Nielsen (MYN) model. Circular maps of the complete mitogenomes were drawn using OrganellarGenomeDRAW (OGDRAW; Greiner et al. 2019).

### 2.5 Phylogeny and Divergence Dating

The same annotation pipeline was used to delimit 13 PCGs and two ribosomal RNA genes in the mitogenomes of *Pergamasus* sp., Parasitidae sp., *Holoparasitus* sp. and *Pergamasus crassipes*. The taxon sampling for phylogenetic reconstruction included 13 ingroup species (or lineages) from the family Parasitidae, with three species (*Stylochyrus rarior* in Ologamasidae, and *Alliphis longicornis* and *Copriphis huainanensis* in Eviphididae) selected as outgroups (Table 1). The 13 PCGs and two rRNA genes were extracted with PhyloSuite v1.2.3 (Xiang et al. 2023). All the genes were aligned in batch mode using MAFFT v7.313 (Katoh and Standley 2013) with the “L‐INS‐I” strategy and the ambiguously aligned regions were removed using the “gappyout” option in trimAl (Capella‐Gutiérrez et al. 2009).

**TABLE 1:** Sources and information used for the phylogenetic analysis.

Family	Subfamily	Species	Size (bp)	Accession number	References
Ologamasidae	—	*Stylochyrus rarior*	14,900	NC_013474	Swafford and Bond (2009)
Eviphididae	—	*Alliphis longicornis*	14,427	PQ611580	Liang et al. (2026)
	—	*Copriphis huainanensis*	14,490	PQ631036	Liang et al. (2026)
Parasitidae	**Parasitinae**	*Cycetogamasus diviortus*	14,450	NC_069643	Liang et al. (2023)
		*Parasitus fimetorum*	14,619	NC_061975	Yang et al. (2022)
		*Parasitus wangdunqingi*	14,541	MK270528	Li et al. (2019)
		*Poecilochirus mrciaki*	14,517	NC_087836	Zhao et al. (2024)
		*Poecilochirus davydovae*	14,489	NC_087857	Zhao et al. (2024)
		*Poecilochirus austroasiaticus*	14,523	PQ535704	This study
		*Poecilochirus subterraneus*	14,490	PQ535705	This study
		*Poecilochirus carabi* BJ1	14,590	PZ188365	This study
		*Poecilochirus carabi* BJ2	14,565	PZ188366	This study
		*Holoparasitus* sp.	14,205	MN622193	Arribas et al. (2020)
	**Pergamasinae**	*Pergamasus* sp.	14,411	MN622219	Arribas et al. (2020)
		*Pergamasus crassipes*	19,144	MN622231	Arribas et al. (2020)
	—	Parasitidae sp.	14,539	MN622232	Arribas et al. (2020)

Phylogenetic relationships were reconstructed using Maximum Likelihood (ML) and Bayesian Inference (BI) based on the concatenated data set of PCGs and rRNAs (PCGR). The best fit substitution models were selected using ModelFinder (Kalyaanamoorthy et al. 2017) using the Bayesian Information Criterion (BIC) (Tables S1 and S2). ML analysis was performed by using IQ‐TREE v2.2.0 (Minh et al. 2020). Nodal support was assessed by using 1000 standard bootstrap replicates. BI analysis was performed in MrBayes v3.2.6 (Ronquist et al. 2012). Two independent runs, each with four Markov chains of 2 million generations were done, sampling every 1000 generations. After checking convergence (average standard deviation of split frequencies < 0.01), the first 25% of samples were discarded as burn‐in and a consensus tree was constructed from the rest of the samples. Final phylogenetic trees were visualized and annotated by using iTOL v6 (Letunic and Bork 2024).

Divergence time estimation for the *P. carabi* complex was performed with the MCMCTREE program implemented in the PAML v4.10.6 package (Reis and Yang 2011; Yang 2007). Analyses were performed with the PCGR dataset (13 protein‐coding genes + 2 rRNA genes) as input, using the BI phylogenetic tree as topological framework. We used a relaxed molecular clock model (clock = 2), and divergence times were estimated with the approximate likelihood method. Molecular clock calibration was performed using two soft bounds based on secondary calibrations and fossil evidence: a soft upper bound of < 238 Ma (‘< 2.38’) on the root node (the MRCA of Ologamasidae, Eviphididae, and Parasitidae) to ensure it is younger than the crown origin of Mesostigmata (Arribas et al. 2020), and a soft minimum constraint of > 44 Ma (‘> 0.44’) on the crown node of Parasitidae based on the oldest Baltic amber fossil (Dunlop and Selden 2009). We modeled the nucleotide substitution process under the GTR + Γ model (model = 7, ncatG = 4), with the ambiguous sites kept (cleandata = 0). MCMC parameters were set as: burnin = 50,000, sampfreq = 5, nsample = 20,000, posterior samples retained. Then, the final divergence time estimates were parsed and drawn with two R packages: the treeio (Wang et al. 2020) package to import MCMCTree output trees, and the ggtree (Yu et al. 2017) package for tree visualization and annotation.

### 2.6 Multi‐Criterion Species Delimitation

Candidate cryptic species diversity within *Poecilochirus* was evaluated using two distinct computational approaches: the tree‐based multi‐rate Poisson Tree Processes (mPTP; Kapli et al. 2017) and the distance‐based Assemble Species by Automatic Partitioning (ASAP; Puillandre et al. 2021). The species delimitation with mPTP was performed on the ML tree with the multi‐rate Poisson Tree Processes model. *Alliphis longicornis*, *Copriphis huainanensis*, and *Stylochyrus rarior* were used as outgroups and automatically removed during the delimitation. The minimum branch length was set to 0.0001, and the significance of the delimitation was evaluated using a likelihood ratio test between the null model and the best‐fit model. For the ASAP analysis, we built a genetic distance matrix based on the concatenated PCGR dataset with the simple distance model. The algorithm heuristically searched for all possible partitions with 1000 repetitions. We selected the partitioning scheme for discriminating between interspecific and intraspecific genetic gaps based on the ranked ASAP scores and a *p*‐value threshold of < 0.01.

## 3 Results

### 3.1 Mitogenomic Organization and Features of Poecilochirus

As other members of the genus *Poecilochirus* (Zhao et al. 2024), the mitogenomes of the four new populations sequenced (*P. austroasiaticus*, *P. subterraneus* , *P. carabi* BJ1, and *P. carabi* BJ2) are also circular double‐stranded DNA molecules (Figure 1). The mitogenomes of the two *P. carabi* populations are the largest, 14,590 bp and 14,565 bp, respectively, and that of *P. austroasiaticus* (14,523 bp) is a little longer than that of *P. subterraneus* (14,490 bp) (Table 2). All six sequenced mitogenomes (including two sequences from Zhao et al. 2024) contain the usual arthropod gene complement: 13 PCGs, two rRNA genes, 22 transfer RNA (tRNA) genes, and a control region (CR). Up to now, the mitochondrial gene order is completely conserved in all the sequenced *Poecilochirus* species. It is the same as the putative ancestral arthropod arrangement, as for example, the horseshoe crab *Limulus polyphemus* (Lavrov et al. 2000).

**FIGURE 1:**
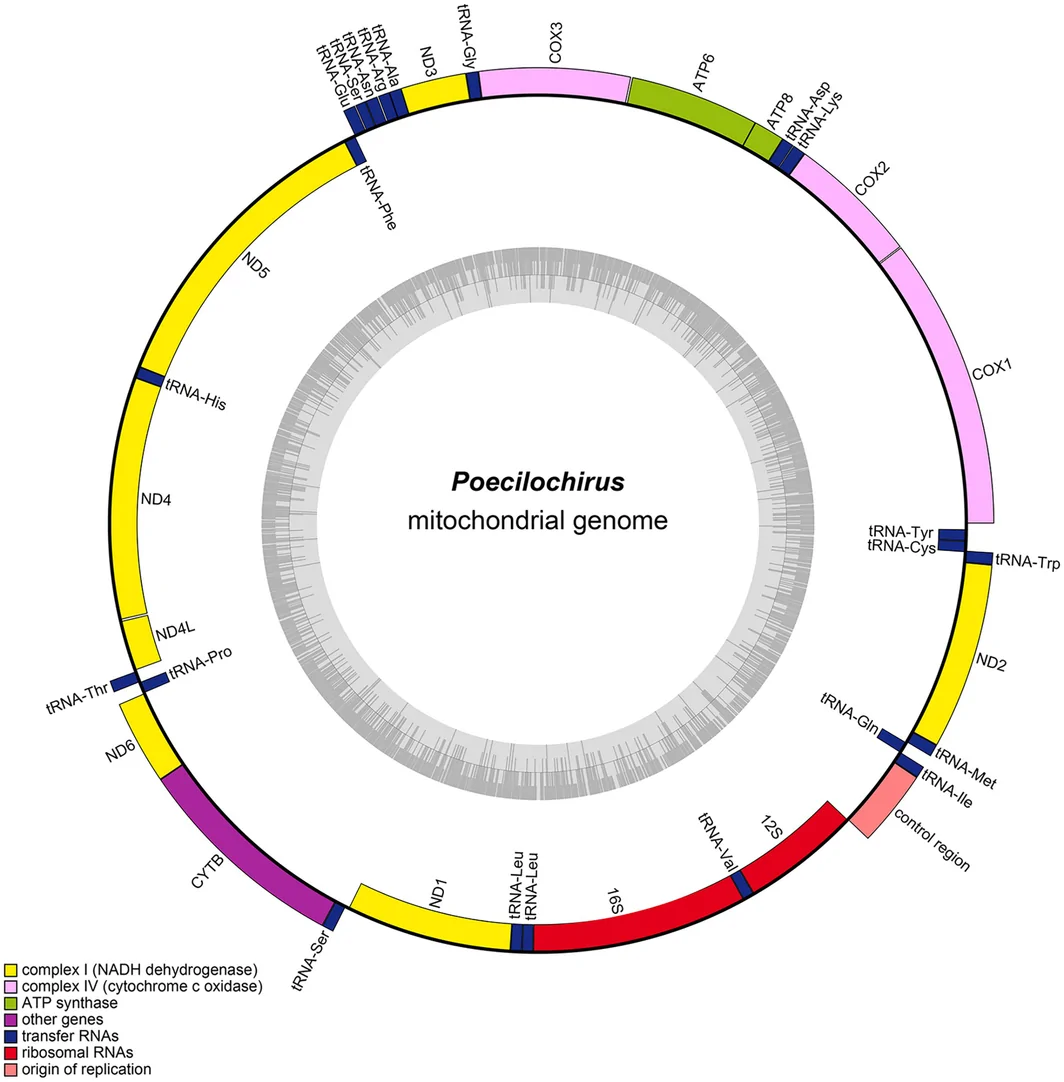
Circular organization of the mitochondrial genome of *Poecilochirus*. Genes on the outside/inside of the circle are transcribed on the (+) and (−) strands, respectively. The inner gray track shows GC content and GC skew.

**TABLE 2:** The mitogenome composition of the six *Poecilochirus* species.

Statistics	Species	Genome	PCGs	rRNA	tRNA	CR
Length (bp)	PD	14,489	10,821	1827	1383	398
	PM	14,517	10,834	1844	1395	398
	PA	14,523	10,831	1842	1381	408
	PS	14,490	10,818	1826	1380	399
	PC 1	14,590	10,846	1864	1387	415
	PC 2	14,565	10,859	1841	1387	415
A + T%	PD	70.08	68.41	75.37	73.32	79.90
	PM	67.81	66.09	73.48	71.33	77.13
	PA	69.74	68.34	75.57	72.27	73.03
	PS	71.86	70.58	75.74	74.42	79.70
	PC 1	73.08	72.28	76.93	73.11	77.11
	PC 2	71.04	69.65	76.54	72.89	77.35
G + C%	PD	29.92	31.59	24.63	26.68	20.1
	PM	32.19	33.91	26.52	28.67	22.87
	PA	30.26	31.66	24.43	27.73	26.97
	PS	28.14	29.42	24.26	25.58	20.3
	PC 1	26.92	27.72	23.07	26.89	22.89
	PC 2	28.96	30.35	23.46	27.11	22.65
AT‐skew%	PD	0.018	−0.165	0.020	0.045	−0.055
	PM	0.040	−0.173	−0.016	0.063	−0.075
	PA	0.020	−0.162	0.016	0.054	−0.007
	PS	0.011	−0.163	0.018	0.053	−0.006
	PC 1	0.006	−0.163	0.007	0.034	0.019
	PC 2	0.017	−0.178	−0.002	0.039	0.053
GC‐skew%	PD	−0.213	−0.009	0.240	−0.144	−0.075
	PM	−0.270	−0.005	0.317	0.105	−0.099
	PA	−0.198	−0.001	0.244	0.102	0.091
	PS	−0.204	0.002	0.273	0.088	−0.012
	PC 1	−0.219	0.017	0.274	0.126	−0.074
	PC 2	−0.226	0.021	0.259	0.117	−0.064

Abbreviations: PA, *Poecilochirus austroasiaticus*; PC 1, *Poecilochirus carabi* BJ1; PC 2, *Poecilochirus carabi* BJ2; PD, *Poecilochirus davydovae*; PM, *Poecilochirus mrciaki*; PS, *Poecilochirus subterraneus*.

In general, the six mitogenomes of *Poecilochirus* mites have a clear AT‐bias. The overall AT contents are in the range from 67.81% to 73.08% (Table 2). For the CR, the AT contents are 73.03%–79.90% (Table 2). Like most Acari mites (Liu et al. 2026; Luo et al. 2025), all six mitogenomes of *Poecilochirus* have positive AT skews (0.006–0.040) and negative GC skews (−0.270 to −0.198) (Table 2). For the six mitogenomes of *Poecilochirus*, excluding the control region, the largest intergenic spacer (31–40 bp) is between *trnY* and *cox1*, and the longest overlapping region (8 bp) is between *trnW* and *trnC* (Table 3).

**TABLE 3:** Organization of the mitochondrial genome of the six *Poecilochirus* species.

Gene	Strand	Length (bp)	Start codon	Stop codon	Intergenic spacer (bp)
COX1	J	1500	ATT (6)	TAA (6)	3 to 9
COX2	J	679	ATG (6)	T‐ (6)	0
tRNA‐K	J	68			0 to 1
tRNA‐D	J	62–65			0
ATP8	J	162	ATA (4), ATT (4), ATC (1)	TAA (6)	−4 to −1
ATP6	J	666	ATA (6)	TAA (6)	3 to 6
COX3	J	778–780	ATG (6)	T‐ (5), TAA (1)	0
tRNA‐G	J	62–63			0
ND3	J	339	ATT (6)	TAG (5), TAA (1)	−2 to −1
tRNA‐A	J	64			−1 to 0
tRNA‐R	J	60–61			3
tRNA‐N	J	63–65			−3 to 11
tRNA‐S1	J	55–63			0 to 3
tRNA‐E	J	63–66			−1 to 1
tRNA‐F	N	63			0
ND5	N	1678–1681	GTG (4), ATG (2)	T‐ (6)	0
tRNA‐H	N	61–64			−1 to 3
ND4	N	1322–1332	ATT (4), ATA (1), TTG (1)	T‐ (3), TAA (3)	2 to 8
ND4L	N	276–285	ATG (4), ATA (1), ATT (1)	TAA (5), T‐ (1)	1 to 6
tRNA‐T	J	62–63			0
tRNA‐P	N	62–63			2
ND6	J	435–438	ATA (2), ATT (2), ATC (2)	TAA (6)	0
CYTB	J	1114	ATG (6)	T‐ (6)	0
tRNA‐S2	J	62			17 to 18
ND1	N	906–909	GTG (5), ATG (1)	TAA (3), TAG (3)	0
tRNA‐L2	N	62			1 to 3
tRNA‐L1	N	62–63			0
I‐rRNA	N	1179–1196			0
tRNA‐V	N	63			0
s‐rRNA	N	646–669			0
CR	J	398–415			0
tRNA‐I	J	63–66			−3
tRNA‐Q	N	63–65			−2 to 3
tRNA‐M	J	63–64			0
ND2	J	961–963	ATT (4), ATC (2)	TAA (5), T‐ (1)	−2 to 0
tRNA‐W	J	63–65			−8
tRNA‐C	N	61–63			0
tRNA‐Y	N	62–63			31 to 40

All four newly sequenced *Poecilochirus* mitogenomes encode the full complement of 22 tRNA genes. As reported previously by Zhao et al. (2024), only the *trnS1* (AGN) gene does not have the D‐arm, while the rest of the 21 tRNAs fold into the canonical cloverleaf secondary structure (Figures S1–S4). Moreover, non‐canonical base‐pair mismatches were also found in the tRNA genes of all four taxa. In addition to the standard Watson‐Crick pairs (A‐U and G‐C), the following mismatches were found: 22 in *P. austroasiaticus* (types: U‐U, G‐A, U‐G, and A‐C); 17 in *P. subterraneus* (U‐U, U‐G, and G‐A); 23 in *P. carabi* BJ1 (U‐U, U‐G, and G‐A); and 22 in *P. carabi* BJ2 (U‐U, U‐G, and G‐A).

### 3.2 Codon Usage and RSCU Preferences

The total lengths of all PCGs of the six *Poecilochirus* mitogenomes are 10,818–10,859 bp, the total length of PCGs of *P. carabi* BJ2 is the longest among all the *Poecilochirus* mitogenomes, and the total length of PCGs of *P. carabi* BJ1 is the second (Table 2). In all the *Poecilochirus* mitogenomes, *nad5* is the longest PCG (1678–1681 bp), while *atp8* is the shortest in all the taxa (162 bp) (Table 3 and Tables S3–S8). Besides the usual ATN start codons (ATA, ATG, and ATT), the rare GTG start codon is also found in all six mitogenomes (Table 4). For the termination signals, both the complete stop codons (TAA and TAG) and the incomplete stop codon (T) were found in the CDS of all the *Poecilochirus* taxa (Table 3). The presence of the truncated T stop codon is a conserved feature widely found in other acarine mites (Liang et al. 2023; Liu et al. 2024; Wu et al. 2019). These incomplete termination signals are usually resolved post‐transcriptionally via polyadenylation to form the canonical TAA stop codon (Huang et al. 2022; Yang et al. 2023b).

**TABLE 4:** Summary of codon usage, start, and stop codons of the 13 PCGs in the mitogenomes of six *Poecilochirus* species.

Species	Total codons	Most frequent AA (count)	Start codons (gene count)	Stop codons (gene count)
*P. davydovae*	3606	Leu (529), Phe (333), Ser (333)	ATT (4), ATG (4), ATA (2), GTG (2), ATC (1)	TAA (7), T (5), TAG (1)
*P. mrciaki*	3609	Leu (524), Ser (341), Phe (335)	ATT (5), ATG (4), ATA (2), GTG (2)	TAA (7), T (4), TAG (2)
*P. austroasiaticus*	3609	Leu (509), Ser (338), Phe (320)	ATG (5), ATT (3), ATA (3), GTG (1), ATC (1)	TAA (8), T (4), TAG (1)
*P. subterraneus*	3604	Leu (514), Phe (334), Ser (334)	ATG (5), ATT (4), ATA (2), GTG (1), TTG (1)	TAA (6), T (6), TAG (1)
*P. carabi* BJ1	3614	Leu (530), Ile (334), Ser (329)	ATT (4), ATG (4), ATA (3), GTG (1), ATC (1)	TAA (7), T (4), TAG (2)
*P. carabi* BJ2	3614	Leu (535), Ser (335), Ile (330)	ATT (5), ATG (3), GTG (2), ATC (2), ATA (1)	TAA (8), T (4), TAG (1)

The relative synonymous codon usage (RSCU) analysis allowed us to observe the codon preference pattern that is specific to the lineage in the mitogenomes from the six *Poecilochirus* species (Figure 2 and Tables S9–S14). All species had a strong preference for the AT‐rich codons; codons ending in A or U (UUA, AUU, and UUU) had a high RSCU value (shading in red/orange), whereas the GC‐rich codons (CGC, CGG, and UCG) were always underused (blue). The distribution corresponds to the high AT nucleotide composition for these mitogenomes. Even if the directional bias is shared, there is interspecific variation in the intensity of the codon use: *P. carabi* (BJ1 and BJ2) has a much higher RSCU value for some of the AT‐rich codons (UUA and UCU) than it does in the other four species (*P. mrciaki*, *P. davydovae*, *P. austroasiaticus*, and *P. subterraneus* ). The dendrogram, which is constructed from Euclidean distances of RSCU profiles, clustered the species in a way that is generally consistent with the established taxonomic lineages. All of these results indicate that the usage of the mitochondrial codon in this genus is determined by the shared evolutionary constraint, such as the AT‐biased mutational pressure, but also reflects the little lineage‐specific divergence in the usage intensity.

**FIGURE 2:**
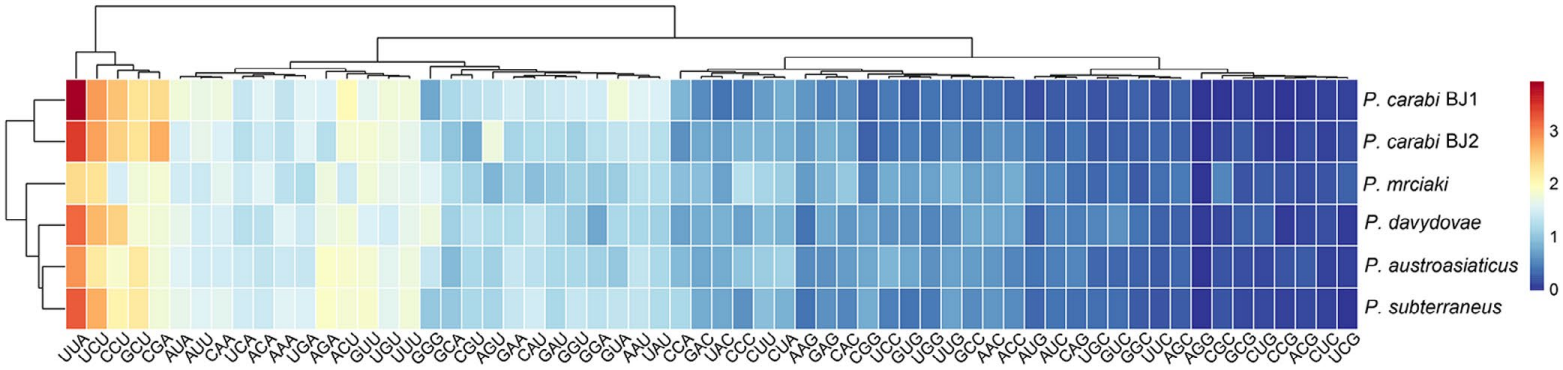
Relative synonymous codon usage (RSCU) in the mitogenomes of *Poecilochirus* mites. Codons (x‐axis) and taxa (y‐axis) are grouped by hierarchical clustering based on RSCU values. The color gradient indicates codon usage frequency, ranging from low (blue, RSCU = 0) to high (red, RSCU > 3).

### 3.3 Sequence Variation and Genetic Divergence

The pairwise *Ka/Ks* ratios for all 13 PCGs across *Poecilochirus* species were consistently below 1 (0.00–0.27; Figure 3), indicating ubiquitous purifying selection across the mitogenome. Among the 13 PCGs, *nad4l* and *atp8* exhibited relatively higher *Ka/Ks* ratios, whereas the cox gene family (*cox1–3*) and cytb displayed the lowest values (*Ka/Ks* < 0.05), reflecting strong functional constraints on core respiratory subunits.

**FIGURE 3:**
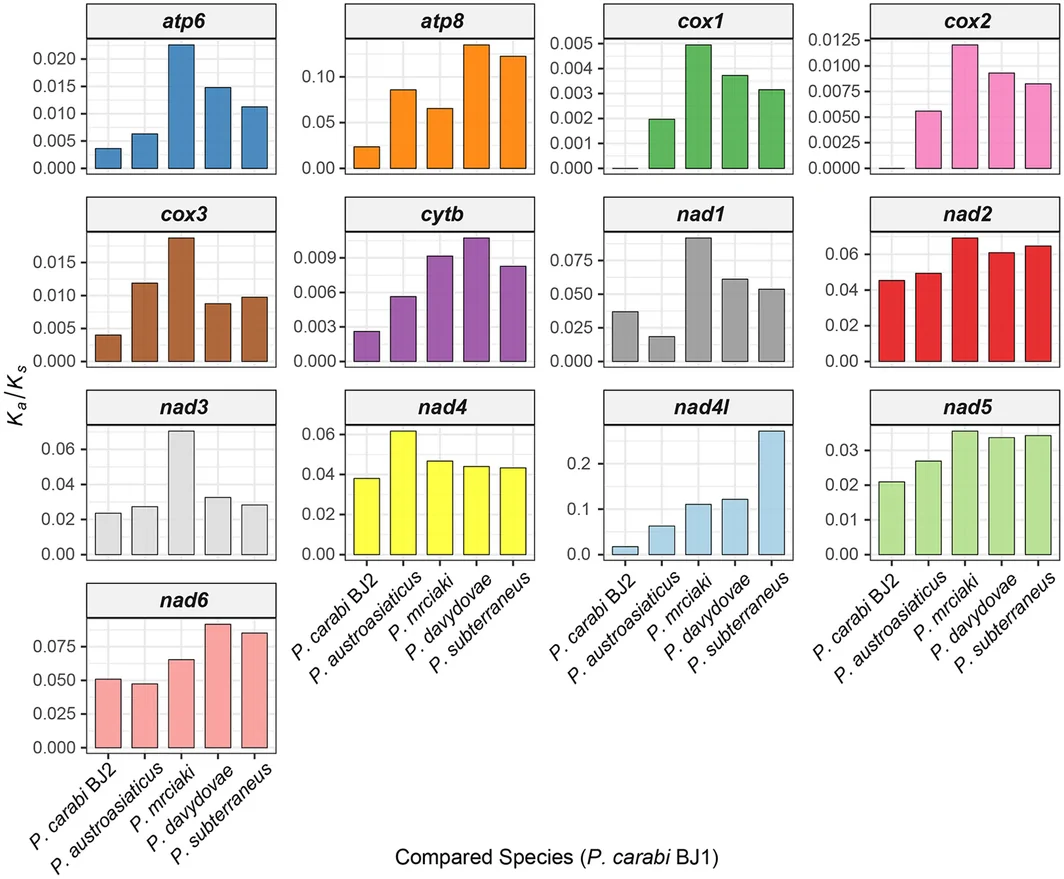
*Ka/Ks* ratios of 13 mitochondrial protein‐coding genes in *Poecilochirus* species relative to *P. carabi* BJ1. The x‐axis shows the five taxa compared against the reference (*P. carabi* BJ1), and the y‐axis indicates the *Ka/Ks* ratio.

Sliding window analysis revealed high spatial heterogeneity in nucleotide diversity (*π*) across the *Poecilochirus* mitogenomes, with a genome‐wide average of 0.197 (Figure 4). The diversity was non‐randomly distributed, identifying distinct hypervariable hotspots within *nad2* (peak *π* = 0.395), *nad5* (peak *π* = 0.392), and *nad6* (peak *π* = 0.386). In contrast, rRNA genes (*16S* and *12S*) and *cox1* showed lower nucleotide diversity (mean *π* = 0.171 for *cox1*). The hypervariable regions in *nad2* and *nad5* provide candidate high‐resolution markers for future species delimitation and population genetics in *Poecilochirus*.

**FIGURE 4:**
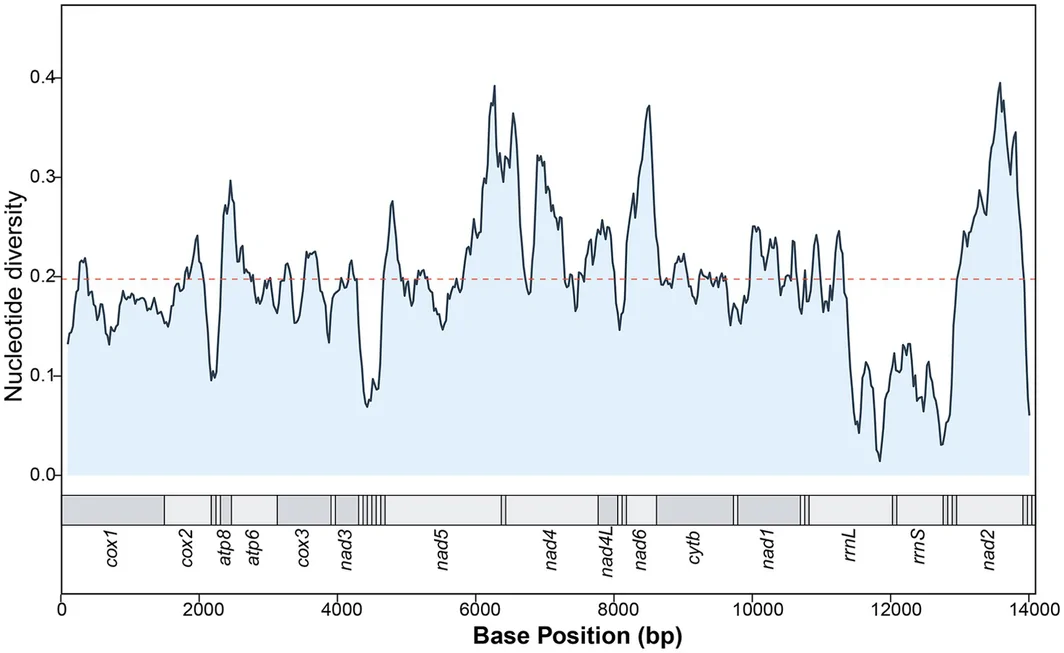
Nucleotide diversity analysis of the *Poecilochirus* mitochondrial genomes. The red dashed line represents the mean value. The x‐axis indicates the alignment base position (bp), and the y‐axis represents nucleotide diversity (*π*).

The pairwise genetic distance analysis showed different degrees of inter‐ and intraspecific differentiation in the genus *Poecilochirus* (Figure 5). For the *cox1* gene (Figure 5A), the genetic distance varies from 0.361 to 1.097; the highest divergence is between *P. mrciaki* and *P. carabi* BJ1 (1.097). The genetic distance between BJ1 and BJ2 within the *P. carabi* species complex is 0.409. This value is quite high for a regular intraspecific level. This potentially reflects deep historical isolation or unrecognized cryptic divergence, which is not yet recognized or deep geographical isolation. The combined analysis of 13 PCGs (Figure 5B) shows that the range of genetic distance is more constrained (0.229–0.810), and the maximum divergence is between *P. mrciaki* and *P. davydovae* (0.810). Under this multi‐locus marker, the distance between *P. carabi* BJ1 and BJ2 is decreased to 0.276, but this is still an indication that they are different genetic lineages. Both the data sets found *P. davydovae* and *P. subterraneus* have the lower interspecific distance (0.229) and it can be regarded as that they have a recent common ancestor. Compared to the single *cox1* marker, these estimates by the 13 PCGs were generally lower and more concentrated. This means that the integration of more mitochondrial PCGs can eliminate the estimation bias caused by the heterogeneous selective pressure or stochastic drift within the individual gene, and it is a better way to check the species differentiation.

**FIGURE 5:**
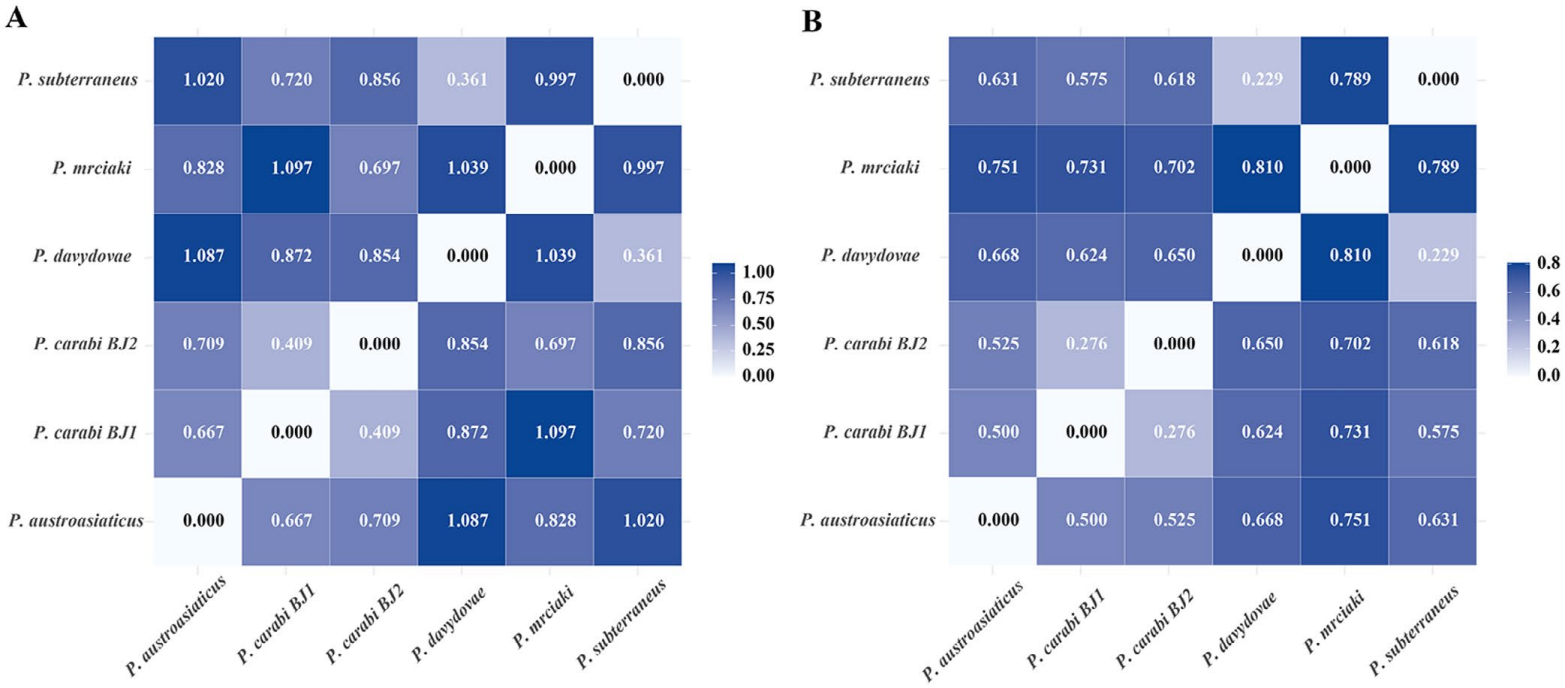
Comparative analysis of interspecific genetic distances within the genus *Poecilochirus*. (A) Pairwise genetic distances based on *cox1* gene sequences; (B) Pairwise genetic distances based on concatenated sequences of 13 PCGs.

### 3.4 Phylogenetic Reconstruction and Taxonomic Implications

The phylogenetic relationships reconstructed by using ML and BI by means of a concatenated mitogenomic data set (13 PCGs and 2 rRNA genes) are shown in Figures S5 and S6 for the genus *Poecilochirus*. Both algorithms gave rise to rather congruent topologies, but differences were found for the nodal support for the several lineages. In both the trees, shows that *Poecilochirus* is paraphyletic and the species of the genus *Parasitus* is nested in the *Poecilochirus* clade, which divides the genus into two main lineages. *P. mrciaki* is basally placed with respect to the rest of the ingroup taxa (ML = 97%, BI = 1.0). In the first lineage, the *P. subterraneus* and *P. davydovae* form a very strong sister group (ML = 100%, BI = 1.0). In the second lineage, the *P. austroasiaticus* is clustering with the *P. carabi* species complex (BJ1 and BJ2); this node has high posterior probability in the BI analysis (BI = 0.982) but only weak bootstrap support in the ML tree (ML = 44%). It seems to be a topologically uncertain node, due to the differences in the methods for resolving short internal branches. For the *P. carabi* BJ1/BJ2 clade, we had the maximum nodal support (ML = 100%, BI = 1.0), but the high pairwise genetic distance reported above (*cox1* = 0.409) suggests the high cryptic divergence in this nominal species. The interdigitated phylogenetic placement of the *Parasitus* and *Poecilochirus* also shows that the actual genus boundaries, which are based on morphology, do not fully reflect their molecular evolutionary history, which requires an integrative taxonomic reassessment of these groups.

### 3.5 Ancient Divergence Times and Species Delimitation

The use of MCMCTREE for Bayesian divergence time estimation also showed an ancient evolutionary history for the genus *Poecilochirus* and the related taxa (Figure 6). The root of the investigated clade, which includes members of Parasitidae, Eviphididae and Ologamasidae, is dated in the Triassic at 223.7 Mya (95% HPD: 179.3–256.8 Mya). The radiation in Parasitinae mainly took place in the Jurassic and Cretaceous. *P. mrciaki* diverges from the core at 140.1 Mya (95% HPD: 106.1–175.1 Mya). At the Early Cretaceous boundary (~129.0 Mya), the core group split into two major lineages. In Lineage A, which includes *P. subterraneus* and *P. davydovae*, the MRCA is at 39.0 Mya (95% HPD: 21.1–60.9 Mya) during the Eocene. In Lineage B, which is intergeneric in nesting of *Parasitus* in *Poecilochirus*, the divergence between *P. austroasiaticus* and the *P. carabi* lineages was dated at 99.9 Mya (95% HPD: 71.7–127.7 Mya). The divergence between *P. carabi* BJ1 and BJ2 was estimated at 52.4 Mya (95% HPD: 30.1–76.0 Mya). The divergence time within this species complex predates the split between the morphologically recognized species *P. subterraneus* and *P. davydovae* (39.0 Mya). These temporal data in combination with a high genetic distance (*cox1* = 0.409), strongly support the status of *P. carabi* BJ1 and BJ2 as distinct candidate cryptic species. Species delimitation based on mPTP and ASAP also consistently partitioned them into distinct candidate molecular operational taxonomic units (MOTUs). mPTP however yields a more conservative delimitation for the ingroup *Pergamasus*/*Holoparasitus* clade.

**FIGURE 6:**
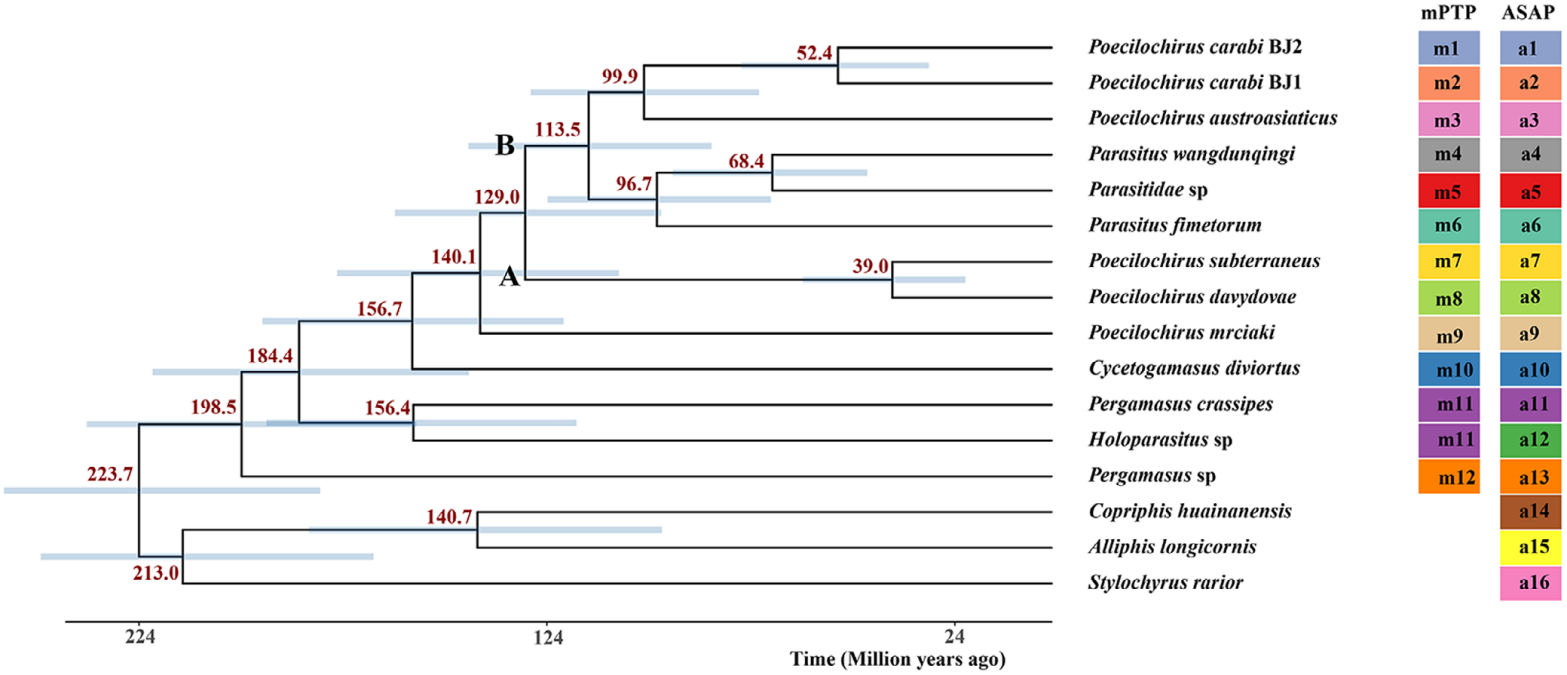
Bayesian divergence time estimation phylogeny of *Poecilochirus* and related lineages. Red numbers at nodes indicate mean divergence times (in million years ago, Ma), with blue horizontal bars representing 95% highest posterior density (HPD) intervals. Columns on the right indicate species delimitation results from mPTP and ASAP analyses. Distinct colors and alphanumeric labels (m1‐m12 for mPTP, a1‐a16 for ASAP) denote unique MOTUs.

## 4 Discussion

### 4.1 Mitogenomic Characteristics and Evolutionary Constraints

As with most mesostigmatid species, the mitogenomes of the genus *Poecilochirus* have also shown significant AT bias (Luo et al. 2025; Yuan et al. 2024). The compositional profile of the mitogenome directly shapes its codon usage. RSCU shows that all lineages always prefer the codon ending with A or U (UUA, AUU, and UUU), which is close to the underlying mutational pressure (Zhang et al. 2025). All 13 PCGs showed *Ka/Ks* < 1 across interspecific comparisons, reflecting the conserved functional constraints typical of the mitochondrial oxidative phosphorylation machinery across the family Parasitidae (Zhao et al. 2024). Despite overall sequence conservation, the sliding window analysis revealed marked spatial heterogeneity in nucleotide diversity (*π*), highlighting *nad2* (peak *π* = 0.395) and *nad5* (peak *π* = 0.392) as two distinct nucleotide variation hotspots. Compared to traditionally used but highly conserved markers such as *cox1*, these hypervariable *nad2* and *nad5* segments represent highly promising, high‐resolution molecular markers for future species delimitation, cryptic lineage identification, and population genetics within this morphologically conservative genus.

### 4.2 Ancient Eocene Divergence in the *P. carabi* Complex

The divergence between the *P. carabi* BJ1 and BJ2 has occurred during the Eocene, around 52.4 Ma (95% HPD: 30.1–76.0 Ma). The divergence here may be related to host evolution and paleoenvironmental changes, since it roughly falls during the early diversification phase of their main host, burying beetles (*Nicrophorus* spp.) (Sikes and Venables 2013). The Paleocene‐Eocene Thermal Maximum promoted the expansion of temperate forests and the diversification of small mammalian taxa, which probably could have increased the availability of carrion resources (Büchner et al. 2024; Gingerich 2006). This can provide an ecological context for the evolution of both hosts and their associated mites. Climatic fluctuations and regional geographic changes during the Eocene–Oligocene transition probably created dispersal barriers for the host populations, which could have facilitated an initial allopatric divergence in the phoretic mites that are tightly associated with them (Botté et al. 2026). The long term maintenance of this deep genetic divergence is mostly due to the high host specificity and extremely limited autonomous dispersal capacity of *Poecilochirus* mites (Müller and Schwarz 1990; Nehring et al. 2017). As obligate phoretic mites, their dispersal and reproductive activities strictly depend on the life history of specific beetle hosts; this ecological dependency restricts the gene flow among different geographic populations or host ecotypes (Canitz et al. 2022; Schwarz 1996; Sun and Kilner 2024). Combined with their reproductive preference for carrion microhabitats and possible differences in the phoretic timing, these ecological and behavioral traits provide a mechanistic explanation for the retention of deep mitochondrial lineage divergence, even if relatively morphologically conservative (Canitz et al. 2022; Sun and Kilner 2024). Overall, the Eocene divergence history and the co‐occurrence of BJ1 and BJ2 on the same beetle host indicate a scenario of historical allopatric divergence followed by secondary contact. These findings are corroborated by the molecular evidence and support recognizing BJ1 and BJ2 as candidate independent evolutionary lineages within the *P. carabi* species complex, providing a temporal evolutionary perspective for their future taxonomic delimitation.

### 4.3 Taxonomic Resolution of the *P. carabi* Complex

Molecular phylogenetic and population genetic evidence suggest that the traditionally recognized *P. carabi* is composed of several cryptic lineages that show a large genetic divergence (Canitz et al. 2022). In this paper, both mPTP and ASAP analyses consistently separated BJ1 and BJ2 as two distinct candidate MOTUs, providing mitogenomic support for cryptic diversity within this nominal taxon. Since adult *Poecilochirus* specimens are seldom recovered in field surveys, the present taxonomy is therefore strongly based on deutonymphal characters, in particular on sternal shield morphology, dorsal shield reticulation, and dorsal setal arrangement (Hyatt 1980; Ma 2005; Mašán 1999). However, these traits show a large interspecific overlap and do not set up stable diagnostic boundaries. Thus, if one would identify only by morphology, the true species diversity and the ecological complexity of the group would be severely underestimated (Canitz et al. 2022; Schwarz 1996). Because mtDNA is a single maternal locus, high mitogenomic divergence alone cannot rule out incomplete lineage sorting, introgression, or mitochondrial capture. Moreover, because each lineage is represented here by a single mitogenomic assembly, within‐lineage variation cannot be evaluated. Therefore, lineages BJ1 and BJ2 remain candidate cryptic species. Formal taxonomic description will require integrative evidence from nuclear markers, detailed morphology, host specificity, and ecological traits. In forensics, we know that *Poecilochirus* mites are used routinely to track the succession stages of a carrion and to estimate the post mortem interval (PMI) (Bala and Singh 2019; Ondrejková et al. 2021; Saloña‐Bordas and Perotti 2014). In the case that the cryptic lineages of the above complex differ in the developmental duration or in environmental tolerances, a treatment of all as only one taxon could introduce a big uncertainty into the models of the PMI estimation (Fuentes‐Lopez et al. 2021). Therefore, we advise that the forensic analyses of the mite samples used for the temporal inference should be supplemented with a molecular validation (e.g., DNA barcoding) (Alejandra Perotti et al. 2009; Boehme et al. 2012). The forensics reports should describe explicitly the identification methodology and the reference databases used, in order to have a greater rigor, transparency and reproducibility in the PMI estimates (Abbasi 2025; Amendt et al. 2007).

### 4.4 Implications for the Systematics of Parasitidae

Our phylogenetic analyses showed that the subfamilies Parasitinae and Pergamasinae were not recovered as reciprocally monophyletic; in fact, their topologies are mixed. In addition, the genus *Poecilochirus* was recovered as paraphyletic, with *Parasitus* nested within the *Poecilochirus* clade (Zhao et al. 2024). While the morphological traits used traditionally to delimit and distinguish parasitid genera have been repeatedly reinterpreted, an integrative revision based on multiple lines of evidence is warranted (Hrúzová and Fenďa 2018). The marked blurring of the generic boundary between *Poecilochirus* and *Parasitus* shows clearly that a morphology‐based approach is limited. However, given the weak bootstrap support at some internal nodes and the single‐locus nature of mitogenomics, formal taxonomic revisions (such as redefining generic boundaries) should be approached with caution and await independent validation from nuclear phylogenomics. This preliminary molecular‐morphological discordance highlights the challenge of defining genera solely by morphological characters and underscores the necessity of integrating multi‐locus nuclear data alongside complete mitogenomes as complementary lines of evidence in parasitid systematics (Canitz et al. 2022; Schlick‐Steiner et al. 2010; Bolbat et al. 2022). Given the still‐limited genomic coverage across the Parasitidae, future work should therefore substantially increase the taxon sampling to include genera and species that are not sequenced yet in a comprehensive way. By integrating densely sampled molecular phylogenies with explicit models of morphological character evolution (Parins‐Fukuchi 2018; Scotland et al. 2003), one can establish a new classification framework for the Parasitidae that at the same time captures patterns of morphological variation and the history of the evolution of the genome.

## 5 Conclusion

The present comparative mitogenomic study provides important insights into the phylogeny and cryptic diversity of the genus *Poecilochirus*. The main results are as follows. The mitogenomes of *Poecilochirus* are very conserved in structure and the gene order is the same as that of the arthropod ancestor. The whole mitogenome is AT‐biased and all 13 PCGs are under purifying selection. The *nad2* and *nad5* are found to be hypervariable hotspots and can be used as the molecular markers for the species delimitation of the genus. The molecular phylogeny shows that *Poecilochirus* is paraphyletic and *Parasitus* is nested in it; the monophyly of the subfamilies Parasitinae and Pergamasinae is not supported. Although the mitochondrial gene tree recovered a non‐monophyletic *Poecilochirus*, formal generic and subfamily revisions should await validation from multi‐locus nuclear phylogenomics. The *cox1* genetic distance between *P. carabi* BJ1 and BJ2 reaches 0.409, and their divergence time is estimated at about 52.4 Ma, which is before the divergence of some of the morphologically recognized species. Both mPTP and ASAP analyses delimited them as distinct MOTUs, providing mitogenomic support for candidate cryptic species within the *P. carabi* complex. The host‐mediated isolation since the Eocene may be an important mechanism to drive the cryptic divergence in this group. For forensic applications, we suggest that the mite samples used for the postmortem interval estimation should be added to the DNA barcoding for the identification of the accuracy and the reliability of the PMI estimate. This study provides important mitogenomic resources to the genus *Poecilochirus*, and it should lay the molecular base for the coming integrative taxonomic revisions and the research of the cryptic species diversity. In future work, the taxon sampling across the Parasitidae should be substantially expanded, and the nuclear gene data as well as the explicit model of the morphological character evolution should be integrated, and thus the classification could be established for the family.

## Author Contributions

**Lang Liang:** data curation (lead), investigation (lead), methodology (lead), resources (lead), writing – original draft (lead). **Pei‐Yue Zhao:** data curation (equal), investigation (equal). **Hu‐Die He:** investigation (equal), methodology (equal). **Tian‐Ci Yi:** project administration (lead), supervision (lead), writing – review and editing (lead). **Dao‐Chao Jin:** project administration (equal), supervision (equal), writing – review and editing (equal).

## Funding

This work was supported by Science and Technology Program of China Tobacco Corporation, 110202401017. National Science and Technology Basic Resources Survey Special Project, 072607060. National Natural Science Foundation of China, 32160118, 32470478.

## Conflicts of Interest

The authors declare no conflicts of interest.

## Supporting information


**Table S1:** The best partitions scheme and models for ML.
**Table S2:** The best partitions scheme and models for BI.
**Table S3:** Organization of the mitochondrial genome of *Poecilochirus davydovae*.
**Table S4:** Organization of the mitochondrial genome of *Poecilochirus mrciaki*.
**Table S5:** Organization of the mitochondrial genome of *Poecilochirus austroasiaticus*.
**Table S6:** Organization of the mitochondrial genome of *Poecilochirus subterraneus*.
**Table S7:** Organization of the mitochondrial genome of *Poecilochirus carabi* BeiJ_1.
**Table S8:** Organization of the mitochondrial genome of *Poecilochirus carabi* BeiJ_2.
**Table S9:** Codon usage for 13 PCGs of the *Poecilochirus davydovae* mitochondrial genome.
**Table S10:** Codon usage for 13 PCGs of the *Poecilochirus mrciaki* mitochondrial genome.
**Table S11:** Codon usage for 13 PCGs of the *Poecilochirus austroasiaticus* mitochondrial genome.
**Table S12:** Codon usage for 13 PCGs of the *Poecilochirus subterraneus* mitochondrial genome.
**Table S13:** Codon usage for 13 PCGs of the *Poecilochirus carabi* BeiJ_1 mitochondrial genome.
**Table S14:** Codon usage for 13 PCGs of the *Poecilochirus carabi* BeiJ_2 mitochondrial genome.
**Figure S1:** Predicted secondary structure of the 22 tRNAs in the *P. austroasiaticus* mitochondrial genome. Watson‐Crick base pairings are indicated by dashes (−): GU pairs are indicated by solid dots: and AU, AA, and UU are indicated by hollow dots.
**Figure S2:** Predicted secondary structure of the 22 tRNAs in the *P. subterraneus* mitochondrial genome. Watson‐Crick base pairings are indicated by dashes (−): GU pairs are indicated by solid dots: and AU, AA, and UU are indicated by hollow dots.
**Figure S3:** Predicted secondary structure of the 22 tRNAs in the *P. carabi* BJ1 mitochondrial genome. Watson‐Crick base pairings are indicated by dashes (−): GU pairs are indicated by solid dots: and AU, AA, and UU are indicated by hollow dots.
**Figure S4:** Predicted secondary structure of the 22 tRNAs in the *P. carabi* BJ2 mitochondrial genome. Watson‐Crick base pairings are indicated by dashes (−): GU pairs are indicated by solid dots: and AU, AA, and UU are indicated by hollow dots.
**Figure S5:** Maximum Likelihood (ML) phylogenetic tree inferred from 13 mitochondrial protein‐coding genes and two rRNA genes.
**Figure S6:** Bayesian Inference (BI) phylogenetic tree inferred from 13 mitochondrial protein‐coding genes and two rRNA genes.

## Data Availability

The genome sequence data supporting this study are publicly available in GenBank (https://www.ncbi.nlm.nih.gov/) under accession numbers PZ188365, PZ188366, PQ535704, and PQ535705.
