# Supplementary material for: Mitogenomic Insights Into the Ancient Split and Cryptic Diversity of the Poecilochirus carabi Complex (Acari: Mesostigmata)
Source: Ecol Evol. 2026 Oct 11;16(10):e74501. doi: 10.1002/ece3.74501 (PMC13654819; doi:10.1002/ece3.74501)
Supplement: Supplementary file 1 [file ECE3-16-e74501-s001.docx]

**Mitogenomic insights into the ancient split and cryptic diversity of the *Poecilochirus carabi* complex (Acari: Mesostigmata)**

Lang Liang^1^, Pei-Yue Zhao^2^, Hu-die He^3^, Dao-Chao Jin^4*^, Tian-Ci Yi^5*^

**Supporting Information**

**Table S1.** The best partitions scheme and models for ML

| **Subset partitions** | **Best model** |
| --- | --- |
| P1: (NAD1) | K3Pu+F+I+G4 |
| P2: (NAD2) | TIM3+F+I+G4 |
| P3: (NAD3) | GTR+F+G4 |
| P4: (NAD4L) | TIM+F+I+G4 |
| P5: (NAD4) | TVM+F+I+G4 |
| P6: (NAD5) | TVM+F+I+G4 |
| P7: (NAD6, ATP6, ATP8, COX2) | TIM+F+I+G4 |
| P8: (COX1) | GTR+F+I+G4 |
| P9: (COX3) | GTR+F+I+G4 |
| P10: (CYTB) | TIM+F+I+G4 |
| P11: (s-rRNA) | TPM3u+F+G4 |
| P12: (I-rRNA) | TVM+F+I+G4 |

**Table S2.** The best partitions scheme and models for BI

| **Subset partitions** | **Best model** |
| --- | --- |
| P1: (NAD1) | GTR+F+I+G4 |
| P2: (NAD2) | GTR+F+I+G4 |
| P3: (NAD3) | GTR+F+G4 |
| P4: (NAD4L) | GTR+F+I+G4 |
| P5: (NAD4) | GTR+F+I+G4 |
| P6: (NAD5) | GTR+F+I+G4 |
| P7: (NAD6, ATP6, ATP8, COX2) | GTR+F+I+G4 |
| P8: (COX1) | GTR+F+I+G4 |
| P9: (COX3) | GTR+F+I+G4 |
| P10: (CYTB) | GTR+F+I+G4 |
| P11: (s-rRNA) | GTR+F+G4 |
| P12: (I-rRNA) | GTR+F+I+G4 |

| **Locus** | **Position** | | **size（bp）** | **Codon** | | | **intergenic nucleotide** | **strand** |
| --- | --- | --- | --- | --- | --- | --- | --- | --- |
|  | **start** | **stop** |  | **start** | **stop** | **anti-codon** |  |  |
| COX1 | 1 | 1500 | 1500 | ATT | TAA |  | 3 | J |
| COX2 | 1504 | 2182 | 679 | ATG | T- |  | 0 | J |
| tRNA-K | 2183 | 2250 | 68 |  |  | CTT | 1 | J |
| tRNA-D | 2252 | 2313 | 62 |  |  | GTC | 0 | J |
| ATP8 | 2314 | 2475 | 162 | ATC | TAA |  | -1 | J |
| ATP6 | 2475 | 3140 | 666 | ATA | TAA |  | 6 | J |
| COX3 | 3147 | 3924 | 778 | ATG | T- |  | 0 | J |
| tRNA-G | 3925 | 3987 | 63 |  |  | TCC | 0 | J |
| ND3 | 3988 | 4326 | 339 | ATT | TAG |  | -2 | J |
| tRNA-A | 4325 | 4388 | 64 |  |  | TGC | -1 | J |
| tRNA-R | 4388 | 4448 | 61 |  |  | TCG | 3 | J |
| tRNA-N | 4452 | 4516 | 65 |  |  | GTT | -2 | J |
| tRNA-S1 | 4515 | 4573 | 59 |  |  | GCT | 0 | J |
| tRNA-E | 4574 | 4638 | 65 |  |  | TTC | 0 | J |
| tRNA-F | 4639 | 4701 | 63 |  |  | GAA | 0 | N |
| ND5 | 4702 | 6379 | 1678 | GTG | T- |  | 0 | N |
| tRNA-H | 6380 | 6441 | 62 |  |  | GTG | 0 | N |
| ND4 | 6442 | 7771 | 1330 | ATT | T- |  | 2 | N |
| ND4L | 7774 | 8049 | 276 | ATG | TAA |  | 6 | N |
| tRNA-T | 8056 | 8117 | 62 |  |  | TGT | 0 | J |
| tRNA-P | 8118 | 8180 | 63 |  |  | TGG | 2 | N |
| ND6 | 8183 | 8617 | 435 | ATA | TAA |  | 0 | J |
| CYTB | 8618 | 9731 | 1114 | ATG | T- |  | 0 | J |
| tRNA-S2 | 9732 | 9793 | 62 |  |  | TGA | 18 | J |
| ND1 | 9812 | 10717 | 906 | GTG | TAA |  | 0 | N |
| tRNA-L2 | 10718 | 10779 | 62 |  |  | TAA | 0 | N |
| tRNA-L1 | 10780 | 10841 | 62 |  |  | TAG | 0 | N |
| I-rRNA | 10842 | 12020 | 1179 |  |  |  | 0 | N |
| tRNA-V | 12021 | 12083 | 63 |  |  | TAC | 0 | N |
| s-rRNA | 12084 | 12731 | 648 |  |  |  | 0 | N |
| CR | 12732 | 13129 | 398 |  |  |  | 0 | J |
| tRNA-I | 13130 | 13192 | 63 |  |  | GAT | -3 | J |
| tRNA-Q | 13190 | 13252 | 63 |  |  | TTG | 2 | N |
| tRNA-M | 13255 | 13318 | 64 |  |  | CAT | 0 | J |
| ND2 | 13319 | 14281 | 963 | ATT | TAA |  | -2 | J |
| tRNA-W | 14280 | 14342 | 63 |  |  | TCA | -8 | J |
| tRNA-C | 14335 | 14395 | 61 |  |  | GCA | 0 | N |
| tRNA-Y | 14396 | 14458 | 63 |  |  | GTA | 31 | N |

**Table S3.** Organization of the mitochondrial genome of *Poecilochirus davydovae*

**Table S4.** Organization of the mitochondrial genome of *Poecilochirus mrciaki*

| **Locus** | **Position** | | **size（bp）** | **Codon** | | | **intergenic nucleotide** | **strand** |
| --- | --- | --- | --- | --- | --- | --- | --- | --- |
|  | **start** | **stop** |  | **start** | **stop** | **anti-codon** |  |  |
| COX1 | 1 | 1500 | 1500 | ATT | TAA |  | 3 | J |
| COX2 | 1504 | 2182 | 679 | ATG | T- |  | 0 | J |
| tRNA-K | 2183 | 2250 | 68 |  |  | CTT | 1 | J |
| tRNA-D | 2252 | 2314 | 63 |  |  | GTC | 0 | J |
| ATP8 | 2315 | 2476 | 162 | ATA | TAA |  | -1 | J |
| ATP6 | 2476 | 3141 | 666 | ATA | TAA |  | 3 | J |
| COX3 | 3145 | 3924 | 780 | ATG | TAA |  | 0 | J |
| tRNA-G | 3925 | 3987 | 63 |  |  | TCC | 0 | J |
| ND3 | 3988 | 4326 | 339 | ATT | TAG |  | -2 | J |
| tRNA-A | 4325 | 4388 | 64 |  |  | TGC | -1 | J |
| tRNA-R | 4388 | 4448 | 61 |  |  | TCG | 3 | J |
| tRNA-N | 4452 | 4514 | 63 |  |  | GTT | -3 | J |
| tRNA-S1 | 4512 | 4574 | 63 |  |  | GCT | 1 | J |
| tRNA-E | 4576 | 4639 | 64 |  |  | TTC | -1 | J |
| tRNA-F | 4639 | 4701 | 63 |  |  | GAA | 0 | N |
| ND5 | 4702 | 6382 | 1681 | GTG | T- |  | 0 | N |
| tRNA-H | 6383 | 6446 | 64 |  |  | GTG | 0 | N |
| ND4 | 6447 | 7776 | 1330 | ATT | T- |  | 2 | N |
| ND4L | 7779 | 8054 | 276 | ATG | TAA |  | 6 | N |
| tRNA-T | 8061 | 8123 | 63 |  |  | TGT | 0 | J |
| tRNA-P | 8124 | 8186 | 63 |  |  | TGG | 2 | N |
| ND6 | 8189 | 8623 | 435 | ATT | TAA |  | 0 | J |
| CYTB | 8624 | 9737 | 1114 | ATG | T- |  | 0 | J |
| tRNA-S2 | 9738 | 9799 | 62 |  |  | TGA | 17 | J |
| ND1 | 9817 | 10725 | 909 | GTG | TAG |  | 0 | N |
| tRNA-L2 | 10726 | 10787 | 62 |  |  | TAA | 0 | N |
| tRNA-L1 | 10788 | 10850 | 63 |  |  | TAG | 0 | N |
| I-rRNA | 10851 | 12046 | 1196 |  |  |  | 0 | N |
| tRNA-V | 12047 | 12109 | 63 |  |  | TAC | 0 | N |
| s-rRNA | 12110 | 12757 | 648 |  |  |  | 0 | N |
| CR | 12758 | 13155 | 398 |  |  |  | 0 | J |
| tRNA-I | 13156 | 13221 | 66 |  |  | GAT | -3 | J |
| tRNA-Q | 13219 | 13282 | 64 |  |  | TTG | -2 | N |
| tRNA-M | 13281 | 13343 | 63 |  |  | CAT | 0 | J |
| ND2 | 13344 | 14306 | 963 | ATT | TAA |  | -2 | J |
| tRNA-W | 14305 | 14369 | 65 |  |  | TCA | -8 | J |
| tRNA-C | 14362 | 14423 | 62 |  |  | GCA | 0 | N |
| tRNA-Y | 14424 | 14486 | 63 |  |  | GTA | 31 | N |

**Table S5.** Organization of the mitochondrial genome of *Poecilochirus austroasiaticus*

| **Locus** | **Position** | | **size（bp）** | **Codon** | | | **intergenic nucleotide** | **strand** |
| --- | --- | --- | --- | --- | --- | --- | --- | --- |
|  | **start** | **stop** |  | **start** | **stop** | **anti-codon** |  |  |
| COX1 | 1 | 1500 | 1500 | ATT | TAA |  | 6 | J |
| COX2 | 1507 | 2185 | 679 | ATG | T- |  | 0 | J |
| tRNA-K | 2186 | 2253 | 68 |  |  | CTT | 1 | J |
| tRNA-D | 2255 | 2317 | 63 |  |  | GTC | 0 | J |
| ATP8 | 2318 | 2479 | 162 | ATA | TAA |  | -4 | J |
| ATP6 | 2476 | 3141 | 666 | ATA | TAA |  | 3 | J |
| COX3 | 3145 | 3922 | 778 | ATG | T- |  | 0 | J |
| tRNA-G | 3923 | 3984 | 62 |  |  | TCC | 0 | J |
| ND3 | 3985 | 4323 | 339 | ATT | TAG |  | -2 | J |
| tRNA-A | 4322 | 4385 | 64 |  |  | TGC | 0 | J |
| tRNA-R | 4386 | 4446 | 61 |  |  | TCG | 3 | J |
| tRNA-N | 4450 | 4512 | 63 |  |  | GTT | 2 | J |
| tRNA-S1 | 4515 | 4571 | 57 |  |  | GCT | 0 | J |
| tRNA-E | 4572 | 4634 | 63 |  |  | TTC | 1 | J |
| tRNA-F | 4636 | 4698 | 63 |  |  | GAA | 0 | N |
| ND5 | 4699 | 6379 | 1681 | ATG | T- |  | 0 | N |
| tRNA-H | 6380 | 6440 | 61 |  |  | GTG | 1 | N |
| ND4 | 6442 | 7770 | 1329 | ATA | TAA |  | 2 | N |
| ND4L | 7773 | 8048 | 276 | ATG | TAA |  | 6 | N |
| tRNA-T | 8055 | 8116 | 62 |  |  | TGT | 0 | J |
| tRNA-P | 8117 | 8178 | 62 |  |  | TGG | 2 | N |
| ND6 | 8181 | 8615 | 435 | ATC | TAA |  | 0 | J |
| CYTB | 8616 | 9729 | 1114 | ATG | T- |  | 0 | J |
| tRNA-S2 | 9730 | 9791 | 62 |  |  | TGA | 18 | J |
| ND1 | 9810 | 10718 | 909 | GTG | TAA |  | 0 | N |
| tRNA-L2 | 10719 | 10780 | 62 |  |  | TAA | 1 | N |
| tRNA-L1 | 10782 | 10843 | 62 |  |  | TAG | 0 | N |
| I-rRNA | 10844 | 12037 | 1194 |  |  |  | 0 | N |
| tRNA-V | 12038 | 12100 | 63 |  |  | TAC | 0 | N |
| s-rRNA | 12101 | 12748 | 648 |  |  |  | 0 | N |
| CR | 12749 | 13156 | 408 |  |  |  | 0 | J |
| tRNA-I | 13157 | 13221 | 65 |  |  | GAT | -3 | J |
| tRNA-Q | 13219 | 13283 | 65 |  |  | TTG | 3 | N |
| tRNA-M | 13287 | 13350 | 64 |  |  | CAT | 0 | J |
| ND2 | 13351 | 14313 | 963 | ATT | TAA |  | -2 | J |
| tRNA-W | 14312 | 14375 | 64 |  |  | TCA | -8 | J |
| tRNA-C | 14368 | 14430 | 63 |  |  | GCA | 0 | N |
| tRNA-Y | 14431 | 14492 | 62 |  |  | GTA | 31 | N |

**Table S6.** Organization of the mitochondrial genome of *Poecilochirus subterraneus*

| **Locus** | **Position** | | **size（bp）** | **Codon** | | | **intergenic nucleotide** | **strand** |
| --- | --- | --- | --- | --- | --- | --- | --- | --- |
|  | **start** | **stop** |  | **start** | **stop** | **anti-codon** |  |  |
| COX1 | 1 | 1500 | 1500 | ATT | TAA |  | 3 | J |
| COX2 | 1504 | 2182 | 679 | ATG | T- |  | 0 | J |
| tRNA-K | 2183 | 2250 | 68 |  |  | CTT | 1 | J |
| tRNA-D | 2252 | 2314 | 63 |  |  | GTC | 0 | J |
| ATP8 | 2315 | 2476 | 162 | ATT | TAA |  | -1 | J |
| ATP6 | 2476 | 3141 | 666 | ATA | TAA |  | 6 | J |
| COX3 | 3148 | 3925 | 778 | ATG | T- |  | 0 | J |
| tRNA-G | 3926 | 3988 | 63 |  |  | TCC | 0 | J |
| ND3 | 3989 | 4327 | 339 | ATT | TAG |  | -2 | J |
| tRNA-A | 4326 | 4389 | 64 |  |  | TGC | -1 | J |
| tRNA-R | 4389 | 4449 | 61 |  |  | TCG | 3 | J |
| tRNA-N | 4453 | 4517 | 65 |  |  | GTT | 0 | J |
| tRNA-S1 | 4518 | 4572 | 55 |  |  | GCT | 2 | J |
| tRNA-E | 4575 | 4639 | 65 |  |  | TTC | 0 | J |
| tRNA-F | 4640 | 4702 | 63 |  |  | GAA | 0 | N |
| ND5 | 4703 | 6380 | 1678 | GTG | T- |  | 0 | N |
| tRNA-H | 6381 | 6442 | 62 |  |  | GTG | 0 | N |
| ND4 | 6443 | 7766 | 1324 | TTG | T- |  | 8 | N |
| ND4L | 7775 | 8050 | 276 | ATG | TAA |  | 6 | N |
| tRNA-T | 8057 | 8118 | 62 |  |  | TGT | 0 | J |
| tRNA-P | 8119 | 8181 | 63 |  |  | TGG | 2 | N |
| ND6 | 8184 | 8618 | 435 | ATA | TAA |  | 0 | J |
| CYTB | 8619 | 9732 | 1114 | ATG | T- |  | 0 | J |
| tRNA-S2 | 9733 | 9794 | 62 |  |  | TGA | 18 | J |
| ND1 | 9813 | 10718 | 906 | ATG | TAA |  | 0 | N |
| tRNA-L2 | 10719 | 10780 | 62 |  |  | TAA | 0 | N |
| tRNA-L1 | 10781 | 10842 | 62 |  |  | TAG | 0 | N |
| I-rRNA | 10843 | 12022 | 1180 |  |  |  | 0 | N |
| tRNA-V | 12023 | 12085 | 63 |  |  | TAC | 0 | N |
| s-rRNA | 12086 | 12731 | 646 |  |  |  | 0 | N |
| CR | 12732 | 13130 | 399 |  |  |  | 0 | J |
| tRNA-I | 13131 | 13193 | 63 |  |  | GAT | -3 | J |
| tRNA-Q | 13191 | 13254 | 64 |  |  | TTG | 2 | N |
| tRNA-M | 13257 | 13320 | 64 |  |  | CAT | 0 | J |
| ND2 | 13321 | 14281 | 961 | ATT | T- |  | 0 | J |
| tRNA-W | 14282 | 14344 | 63 |  |  | TCA | -8 | J |
| tRNA-C | 14337 | 14397 | 61 |  |  | GCA | 0 | N |
| tRNA-Y | 14398 | 14459 | 62 |  |  | GTA | 31 | N |

**Table S7.** Organization of the mitochondrial genome of *Poecilochirus carabi* BeiJ_1

| **Locus** | **Position** | | | **size（bp）** | | **Codon** | | | | | **intergenic nucleotide** | | **strand** |
| --- | --- | --- | --- | --- | --- | --- | --- | --- | --- | --- | --- | --- | --- |
|  | **start** | **stop** | |  |  | **start** | **stop** | | **anti-codon** | |  |  |  |
| COX1 | 1 | 1500 | | 1500 | | ATT | TAA | |  | | 8 | | J |
| COX2 | 1509 | 2187 | | 679 | | ATG | T- | |  | | 0 | | J |
| tRNA-K | 2188 | 2255 | | 68 | |  |  | | CTT | | 0 | | J |
| tRNA-D | 2256 | 2320 | | 65 | |  |  | | GTC | | 0 | | J |
| ATP8 | 2321 | 2482 | | 162 | | ATA | TAA | |  | | -4 | | J |
| ATP6 | 2479 | 3144 | | 666 | | ATA | TAA | |  | | 6 | | J |
| COX3 | 3151 | 3928 | | 778 | | ATG | T- | |  | | 0 | | J |
| tRNA-G | 3929 | 3990 | | 62 | |  |  | | TCC | | 0 | | J |
| ND3 | 3991 | 4329 | | 339 | | ATT | TAG | |  | | -2 | | J |
| tRNA-A | 4328 | 4391 | | 64 | |  |  | | TGC | | 0 | | J |
| tRNA-R | 4392 | 4451 | | 60 | |  |  | | TCG | | 3 | | J |
| tRNA-N | 4455 | 4517 | | 63 | |  |  | | GTT | | 11 | | J |
| tRNA-S1 | 4529 | 4587 | | 59 | |  |  | | GCT | | 3 | | J |
| tRNA-E | 4591 | 4655 | | 65 | |  |  | | TTC | | 0 | | J |
| tRNA-F | 4656 | 4718 | | 63 | |  |  | | GAA | | 0 | | N |
| ND5 | 4719 | 6399 | | 1681 | | ATG | T- | |  | | 0 | | N |
| tRNA-H | 6400 | 6461 | | 62 | |  |  | | GTG | | -1 | | N |
| ND4 | 6461 | 7792 | | 1332 | | ATT | TAA | |  | | 2 | | N |
| ND4L | 7795 | 8079 | | 285 | | ATA | TAA | |  | | 1 | | N |
| tRNA-T | 8081 | 8143 | | 63 | |  |  | | TGT | | 0 | | J |
| tRNA-P | 8144 | 8205 | | 62 | |  |  | | TGG | | 2 | | N |
| ND6 | 8208 | 8645 | | 438 | | ATT | TAA | |  | | 0 | | J |
| CYTB | 8646 | | 9759 | | 1114 | ATG | | T- | |  | | 0 | J |
| tRNA-S2 | 9760 | | 9821 | | 62 |  | |  | | TGA | | 18 | J |
| ND1 | 9840 | | 10748 | | 909 | GTG | | TAG | |  | | 0 | N |
| tRNA-L2 | 10749 | | 10810 | | 62 |  | |  | | TAA | | 3 | N |
| tRNA-L1 | 10814 | | 10876 | | 63 |  | |  | | TAG | | 0 | N |
| I-rRNA | 10877 | | 12071 | | 1195 |  | |  | |  | | 0 | N |
| tRNA-V | 12072 | | 12134 | | 63 |  | |  | | TAC | | 0 | N |
| s-rRNA | 12135 | | 12803 | | 669 |  | |  | |  | | 0 | N |
| CR | 12804 | | 13218 | | 415 |  | |  | |  | | 0 | J |
| tRNA-I | 13219 | | 13282 | | 64 |  | |  | | GAT | | -3 | J |
| tRNA-Q | 13280 | | 13344 | | 65 |  | |  | | TTG | | 2 | N |
| tRNA-M | 13347 | | 13410 | | 64 |  | |  | | CAT | | 0 | J |
| ND2 | 13411 | | 14373 | | 963 | ATC | | TAA | |  | | 0 | J |
| tRNA-W | 14374 | | 14436 | | 63 |  | |  | | TCA | | -8 | J |
| tRNA-C | 14429 | | 14490 | | 62 |  | |  | | GCA | | 0 | N |
| tRNA-Y | 14491 | | 14553 | | 63 |  | |  | | GTA | | 37 | N |

**Table S8.** Organization of the mitochondrial genome of *Poecilochirus carabi* BeiJ_2

| **Locus** | **Position** | | | **size（bp）** | | **Codon** | | | | | **intergenic nucleotide** | | **strand** |
| --- | --- | --- | --- | --- | --- | --- | --- | --- | --- | --- | --- | --- | --- |
|  | **start** | **stop** | |  |  | **start** | **stop** | | **anti-codon** | |  |  |  |
| COX1 | 1 | 1500 | | 1500 | | ATT | TAA | |  | | 9 | | J |
| COX2 | 1510 | 2188 | | 679 | | ATG | T- | |  | | 0 | | J |
| tRNA-K | 2189 | 2256 | | 68 | |  |  | | CTT | | 0 | | J |
| tRNA-D | 2257 | 2320 | | 64 | |  |  | | GTC | | 0 | | J |
| ATP8 | 2321 | 2482 | | 162 | | ATT | TAA | |  | | -4 | | J |
| ATP6 | 2479 | 3144 | | 666 | | ATA | TAA | |  | | 6 | | J |
| COX3 | 3151 | 3928 | | 778 | | ATG | T- | |  | | 0 | | J |
| tRNA-G | 3929 | 3991 | | 63 | |  |  | | TCC | | 0 | | J |
| ND3 | 3992 | 4330 | | 339 | | ATT | TAA | |  | | -1 | | J |
| tRNA-A | 4330 | 4393 | | 64 | |  |  | | TGC | | 0 | | J |
| tRNA-R | 4394 | 4454 | | 61 | |  |  | | TCG | | 3 | | J |
| tRNA-N | 4458 | 4520 | | 63 | |  |  | | GTT | | 2 | | J |
| tRNA-S1 | 4523 | 4581 | | 59 | |  |  | | GCT | | 3 | | J |
| tRNA-E | 4585 | 4650 | | 66 | |  |  | | TTC | | 0 | | J |
| tRNA-F | 4651 | 4713 | | 63 | |  |  | | GAA | | 0 | | N |
| ND5 | 4714 | 6394 | | 1681 | | GTG | T- | |  | | 0 | | N |
| tRNA-H | 6395 | 6456 | | 62 | |  |  | | GTG | | 3 | | N |
| ND4 | 6460 | 7791 | | 1332 | | ATT | TAA | |  | | -7 | | N |
| ND4L | 7794 | 8078 | | 285 | | ATT | TAA | |  | | 2 | | N |
| tRNA-T | 8081 | 8142 | | 62 | |  |  | | TGT | | 0 | | J |
| tRNA-P | 8143 | 8204 | | 62 | |  |  | | TGG | | 2 | | N |
| ND6 | 8207 | 8644 | | 438 | | ATC | TAA | |  | | 0 | | J |
| CYTB | 8645 | 9758 | | 1114 | | ATG | T- | |  | | 0 | | J |
| tRNA-S2 | 9759 | 9820 | | 62 | |  |  | | TGA | | 18 | | J |
| ND1 | 9839 | | 10747 | | 909 | GTG | | TAG | |  | | 0 | N |
| tRNA-L2 | 10748 | | 10809 | | 62 |  | |  | | TAA | | 1 | N |
| tRNA-L1 | 10811 | | 10872 | | 62 |  | |  | | TAG | | 0 | N |
| I-rRNA | 10873 | | 12064 | | 1192 |  | |  | |  | | 0 | N |
| tRNA-V | 12065 | | 12127 | | 63 |  | |  | | TAC | | 0 | N |
| s-rRNA | 12128 | | 12776 | | 649 |  | |  | |  | | 0 | N |
| CR | 12777 | | 13191 | | 415 |  | |  | |  | | 0 | J |
| tRNA-I | 13192 | | 13255 | | 64 |  | |  | | GAT | | -3 | J |
| tRNA-Q | 13253 | | 13317 | | 65 |  | |  | | TTG | | 3 | N |
| tRNA-M | 13321 | | 13384 | | 64 |  | |  | | CAT | | 0 | J |
| ND2 | 13385 | | 14347 | | 963 | ATC | | TAA | |  | | -2 | J |
| tRNA-W | 14346 | | 14408 | | 63 |  | |  | | TCA | | -8 | J |
| tRNA-C | 14401 | | 14462 | | 62 |  | |  | | GCA | | 0 | N |
| tRNA-Y | 14463 | | 14525 | | 63 |  | |  | | GTA | | 40 | N |

**Table S9.** Codon usage for 13 PCGs of the *Poecilochirus davydovae* mitochondrial genome

| **Codon** | **Count** | **RSCU** | **Codon** | **Count** | **RSCU** | **Codon** | **Count** | **RSCU** | **Codon** | **Count** | **RSCU** |
| --- | --- | --- | --- | --- | --- | --- | --- | --- | --- | --- | --- |
| UUU(F) | 282 | 1.69 | UCU(S) | 111 | 2.67 | UAU(Y) | 87 | 1.14 | UGU(C) | 25 | 1.47 |
| UUC(F) | 51 | 0.31 | UCC(S) | 26 | 0.62 | UAC(Y) | 65 | 0.86 | UGC(C) | 9 | 0.53 |
| UUA(L) | 279 | 3.16 | UCA(S) | 54 | 1.3 | UAA(*) | 7 | 1.75 | UGA(W) | 69 | 1.45 |
| UUG(L) | 54 | 0.61 | UCG(S) | 3 | 0.07 | UAG(*) | 1 | 0.25 | UGG(W) | 26 | 0.55 |
| CUU(L) | 82 | 0.93 | CCU(P) | 87 | 2.47 | CAU(H) | 49 | 1.36 | CGU(R) | 18 | 1.33 |
| CUC(L) | 14 | 0.16 | CCC(P) | 26 | 0.74 | CAC(H) | 23 | 0.64 | CGC(R) | 2 | 0.15 |
| CUA(L) | 75 | 0.85 | CCA(P) | 26 | 0.74 | CAA(Q) | 37 | 1.51 | CGA(R) | 24 | 1.78 |
| CUG(L) | 25 | 0.28 | CCG(P) | 2 | 0.06 | CAG(Q) | 12 | 0.49 | CGG(R) | 10 | 0.74 |
| AUU(I) | 233 | 1.48 | ACU(T) | 86 | 1.81 | AAU(N) | 104 | 1.22 | AGU(S) | 52 | 1.25 |
| AUC(I) | 82 | 0.52 | ACC(T) | 35 | 0.74 | AAC(N) | 66 | 0.78 | AGC(S) | 13 | 0.31 |
| AUA(M) | 223 | 1.67 | ACA(T) | 63 | 1.33 | AAA(K) | 72 | 1.58 | AGA(S) | 72 | 1.73 |
| AUG(M) | 44 | 0.33 | ACG(T) | 6 | 0.13 | AAG(K) | 19 | 0.42 | AGG(S) | 2 | 0.05 |
| GUU(V) | 93 | 1.55 | GCU(A) | 73 | 1.84 | GAU(D) | 40 | 1.18 | GGU(G) | 57 | 1.06 |
| GUC(V) | 37 | 0.62 | GCC(A) | 30 | 0.75 | GAC(D) | 28 | 0.82 | GGC(G) | 22 | 0.41 |
| GUA(V) | 72 | 1.2 | GCA(A) | 45 | 1.13 | GAA(E) | 57 | 1.25 | GGA(G) | 42 | 0.78 |
| GUG(V) | 38 | 0.63 | GCG(A) | 11 | 0.28 | GAG(E) | 34 | 0.75 | GGG(G) | 94 | 1.75 |

**Table S10.** Codon usage for 13 PCGs of the *Poecilochirus mrciaki* mitochondrial genome

| **Codon** | **Count** | **RSCU** | **Codon** | **Count** | **RSCU** | **Codon** | **Count** | **RSCU** | **Codon** | **Count** | **RSCU** |
| --- | --- | --- | --- | --- | --- | --- | --- | --- | --- | --- | --- |
| UUU(F) | 271 | 1.62 | UCU(S) | 99 | 2.32 | UAU(Y) | 84 | 1.26 | UGU(C) | 30 | 1.67 |
| UUC(F) | 64 | 0.38 | UCC(S) | 35 | 0.82 | UAC(Y) | 49 | 0.74 | UGC(C) | 6 | 0.33 |
| UUA(L) | 210 | 2.4 | UCA(S) | 62 | 1.45 | UAA(*) | 7 | 1.56 | UGA(W) | 58 | 1.26 |
| UUG(L) | 79 | 0.9 | UCG(S) | 13 | 0.3 | UAG(*) | 2 | 0.44 | UGG(W) | 34 | 0.74 |
| CUU(L) | 101 | 1.16 | CCU(P) | 52 | 1.5 | CAU(H) | 38 | 0.95 | CGU(R) | 15 | 1.11 |
| CUC(L) | 19 | 0.22 | CCC(P) | 45 | 1.29 | CAC(H) | 42 | 1.05 | CGC(R) | 7 | 0.52 |
| CUA(L) | 94 | 1.08 | CCA(P) | 34 | 0.98 | CAA(Q) | 40 | 1.63 | CGA(R) | 25 | 1.85 |
| CUG(L) | 21 | 0.24 | CCG(P) | 8 | 0.23 | CAG(Q) | 9 | 0.37 | CGG(R) | 7 | 0.52 |
| AUU(I) | 221 | 1.45 | ACU(T) | 70 | 1.44 | AAU(N) | 93 | 1.27 | AGU(S) | 37 | 0.87 |
| AUC(I) | 84 | 0.55 | ACC(T) | 39 | 0.8 | AAC(N) | 54 | 0.73 | AGC(S) | 20 | 0.47 |
| AUA(M) | 187 | 1.47 | ACA(T) | 77 | 1.58 | AAA(K) | 61 | 1.33 | AGA(S) | 75 | 1.76 |
| AUG(M) | 68 | 0.53 | ACG(T) | 9 | 0.18 | AAG(K) | 31 | 0.67 | AGG(S) | 0 | 0 |
| GUU(V) | 119 | 1.78 | GCU(A) | 78 | 1.79 | GAU(D) | 35 | 1.03 | GGU(G) | 62 | 1.11 |
| GUC(V) | 28 | 0.42 | GCC(A) | 32 | 0.74 | GAC(D) | 33 | 0.97 | GGC(G) | 14 | 0.25 |
| GUA(V) | 72 | 1.08 | GCA(A) | 55 | 1.26 | GAA(E) | 50 | 1.1 | GGA(G) | 59 | 1.06 |
| GUG(V) | 48 | 0.72 | GCG(A) | 9 | 0.21 | GAG(E) | 41 | 0.9 | GGG(G) | 88 | 1.58 |

**Table S11.** Codon usage for 13 PCGs of the *Poecilochirus austroasiaticus* mitochondrial genome

| **Codon** | **Count** | **RSCU** | **Codon** | **Count** | **RSCU** | **Codon** | **Count** | **RSCU** | **Codon** | **Count** | **RSCU** |
| --- | --- | --- | --- | --- | --- | --- | --- | --- | --- | --- | --- |
| UUU(F) | 279 | 1.74 | UCU(S) | 94 | 2.22 | UAU(Y) | 94 | 1.2 | UGU(C) | 28 | 1.65 |
| UUC(F) | 41 | 0.26 | UCC(S) | 37 | 0.88 | UAC(Y) | 63 | 0.8 | UGC(C) | 6 | 0.35 |
| UUA(L) | 244 | 2.88 | UCA(S) | 61 | 1.44 | UAA(*) | 8 | 1.78 | UGA(W) | 62 | 1.39 |
| UUG(L) | 61 | 0.72 | UCG(S) | 4 | 0.09 | UAG(*) | 1 | 0.22 | UGG(W) | 27 | 0.61 |
| CUU(L) | 92 | 1.08 | CCU(P) | 65 | 1.86 | CAU(H) | 51 | 1.31 | CGU(R) | 16 | 1.21 |
| CUC(L) | 7 | 0.08 | CCC(P) | 35 | 1 | CAC(H) | 27 | 0.69 | CGC(R) | 3 | 0.23 |
| CUA(L) | 94 | 1.11 | CCA(P) | 32 | 0.91 | CAA(Q) | 37 | 1.48 | CGA(R) | 24 | 1.81 |
| CUG(L) | 11 | 0.13 | CCG(P) | 8 | 0.23 | CAG(Q) | 13 | 0.52 | CGG(R) | 10 | 0.75 |
| AUU(I) | 234 | 1.49 | ACU(T) | 93 | 1.86 | AAU(N) | 104 | 1.36 | AGU(S) | 47 | 1.11 |
| AUC(I) | 81 | 0.51 | ACC(T) | 27 | 0.54 | AAC(N) | 49 | 0.64 | AGC(S) | 14 | 0.33 |
| AUA(M) | 223 | 1.6 | ACA(T) | 68 | 1.36 | AAA(K) | 66 | 1.42 | AGA(S) | 81 | 1.92 |
| AUG(M) | 55 | 0.4 | ACG(T) | 12 | 0.24 | AAG(K) | 27 | 0.58 | AGG(S) | 0 | 0 |
| GUU(V) | 115 | 1.85 | GCU(A) | 96 | 2.21 | GAU(D) | 41 | 1.22 | GGU(G) | 66 | 1.22 |
| GUC(V) | 20 | 0.32 | GCC(A) | 30 | 0.69 | GAC(D) | 26 | 0.78 | GGC(G) | 15 | 0.28 |
| GUA(V) | 66 | 1.06 | GCA(A) | 41 | 0.94 | GAA(E) | 60 | 1.38 | GGA(G) | 61 | 1.12 |
| GUG(V) | 47 | 0.76 | GCG(A) | 7 | 0.16 | GAG(E) | 27 | 0.62 | GGG(G) | 75 | 1.38 |

**Table S12.** Codon usage for 13 PCGs of the *Poecilochirus subterraneus* mitochondrial genome

| **Codon** | **Count** | **RSCU** | **Codon** | **Count** | **RSCU** | **Codon** | **Count** | **RSCU** | **Codon** | **Count** | **RSCU** |
| --- | --- | --- | --- | --- | --- | --- | --- | --- | --- | --- | --- |
| UUU(F) | 301 | 1.8 | UCU(S) | 114 | 2.73 | UAU(Y) | 93 | 1.24 | UGU(C) | 31 | 1.63 |
| UUC(F) | 33 | 0.2 | UCC(S) | 20 | 0.48 | UAC(Y) | 57 | 0.76 | UGC(C) | 7 | 0.37 |
| UUA(L) | 278 | 3.25 | UCA(S) | 61 | 1.46 | UAA(*) | 6 | 1.71 | UGA(W) | 73 | 1.55 |
| UUG(L) | 61 | 0.71 | UCG(S) | 1 | 0.02 | UAG(*) | 1 | 0.29 | UGG(W) | 21 | 0.45 |
| CUU(L) | 83 | 0.97 | CCU(P) | 73 | 2.09 | CAU(H) | 54 | 1.46 | CGU(R) | 16 | 1.19 |
| CUC(L) | 8 | 0.09 | CCC(P) | 22 | 0.63 | CAC(H) | 20 | 0.54 | CGC(R) | 3 | 0.22 |
| CUA(L) | 73 | 0.85 | CCA(P) | 42 | 1.2 | CAA(Q) | 40 | 1.63 | CGA(R) | 24 | 1.78 |
| CUG(L) | 11 | 0.13 | CCG(P) | 3 | 0.09 | CAG(Q) | 9 | 0.37 | CGG(R) | 11 | 0.81 |
| AUU(I) | 262 | 1.6 | ACU(T) | 88 | 1.85 | AAU(N) | 113 | 1.34 | AGU(S) | 48 | 1.15 |
| AUC(I) | 65 | 0.4 | ACC(T) | 27 | 0.57 | AAC(N) | 56 | 0.66 | AGC(S) | 10 | 0.24 |
| AUA(M) | 236 | 1.67 | ACA(T) | 69 | 1.45 | AAA(K) | 72 | 1.58 | AGA(S) | 80 | 1.92 |
| AUG(M) | 46 | 0.33 | ACG(T) | 6 | 0.13 | AAG(K) | 19 | 0.42 | AGG(S) | 0 | 0 |
| GUU(V) | 108 | 1.87 | GCU(A) | 86 | 2.22 | GAU(D) | 42 | 1.2 | GGU(G) | 75 | 1.4 |
| GUC(V) | 22 | 0.38 | GCC(A) | 23 | 0.59 | GAC(D) | 28 | 0.8 | GGC(G) | 13 | 0.24 |
| GUA(V) | 78 | 1.35 | GCA(A) | 40 | 1.03 | GAA(E) | 60 | 1.38 | GGA(G) | 71 | 1.33 |
| GUG(V) | 23 | 0.4 | GCG(A) | 6 | 0.15 | GAG(E) | 27 | 0.62 | GGG(G) | 55 | 1.03 |

**Table S13.** Codon usage for 13 PCGs of the *Poecilochirus carabi* BeiJ_1 mitochondrial genome

| **Codon** | **Count** | **RSCU** | **Codon** | **Count** | **RSCU** | **Codon** | **Count** | **RSCU** | **Codon** | **Count** | **RSCU** |
| --- | --- | --- | --- | --- | --- | --- | --- | --- | --- | --- | --- |
| UUU(F) | 292 | 1.78 | UCU(S) | 118 | 2.87 | UAU(Y) | 125 | 1.57 | UGU(C) | 27 | 1.8 |
| UUC(F) | 36 | 0.22 | UCC(S) | 18 | 0.44 | UAC(Y) | 34 | 0.43 | UGC(C) | 3 | 0.2 |
| UUA(L) | 348 | 3.94 | UCA(S) | 58 | 1.41 | UAA(*) | 7 | 1.56 | UGA(W) | 71 | 1.58 |
| UUG(L) | 34 | 0.38 | UCG(S) | 4 | 0.1 | UAG(*) | 2 | 0.44 | UGG(W) | 19 | 0.42 |
| CUU(L) | 60 | 0.68 | CCU(P) | 91 | 2.58 | CAU(H) | 52 | 1.37 | CGU(R) | 18 | 1.33 |
| CUC(L) | 10 | 0.11 | CCC(P) | 18 | 0.51 | CAC(H) | 24 | 0.63 | CGC(R) | 0 | 0 |
| CUA(L) | 72 | 0.82 | CCA(P) | 31 | 0.88 | CAA(Q) | 44 | 1.76 | CGA(R) | 32 | 2.37 |
| CUG(L) | 6 | 0.07 | CCG(P) | 1 | 0.03 | CAG(Q) | 6 | 0.24 | CGG(R) | 4 | 0.3 |
| AUU(I) | 289 | 1.73 | ACU(T) | 92 | 2.03 | AAU(N) | 132 | 1.61 | AGU(S) | 55 | 1.34 |
| AUC(I) | 45 | 0.27 | ACC(T) | 14 | 0.31 | AAC(N) | 32 | 0.39 | AGC(S) | 12 | 0.29 |
| AUA(M) | 243 | 1.81 | ACA(T) | 73 | 1.61 | AAA(K) | 60 | 1.35 | AGA(S) | 64 | 1.56 |
| AUG(M) | 26 | 0.19 | ACG(T) | 2 | 0.04 | AAG(K) | 29 | 0.65 | AGG(S) | 0 | 0 |
| GUU(V) | 93 | 1.65 | GCU(A) | 102 | 2.31 | GAU(D) | 47 | 1.42 | GGU(G) | 81 | 1.46 |
| GUC(V) | 14 | 0.25 | GCC(A) | 16 | 0.36 | GAC(D) | 19 | 0.58 | GGC(G) | 16 | 0.29 |
| GUA(V) | 102 | 1.81 | GCA(A) | 54 | 1.22 | GAA(E) | 67 | 1.47 | GGA(G) | 82 | 1.48 |
| GUG(V) | 16 | 0.28 | GCG(A) | 5 | 0.11 | GAG(E) | 24 | 0.53 | GGG(G) | 43 | 0.77 |

**Table S14.** Codon usage for 13 PCGs of the *Poecilochirus carabi* BeiJ_2 mitochondrial genome

| **Codon** | **Count** | **RSCU** | **Codon** | **Count** | **RSCU** | **Codon** | **Count** | **RSCU** | **Codon** | **Count** | **RSCU** |
| --- | --- | --- | --- | --- | --- | --- | --- | --- | --- | --- | --- |
| UUU(F) | 269 | 1.68 | UCU(S) | 118 | 2.83 | UAU(Y) | 105 | 1.28 | UGU(C) | 27 | 1.74 |
| UUC(F) | 51 | 0.32 | UCC(S) | 17 | 0.41 | UAC(Y) | 59 | 0.72 | UGC(C) | 4 | 0.26 |
| UUA(L) | 306 | 3.44 | UCA(S) | 54 | 1.29 | UAA(*) | 8 | 1.78 | UGA(W) | 71 | 1.58 |
| UUG(L) | 56 | 0.63 | UCG(S) | 7 | 0.17 | UAG(*) | 1 | 0.22 | UGG(W) | 19 | 0.42 |
| CUU(L) | 79 | 0.89 | CCU(P) | 85 | 2.45 | CAU(H) | 46 | 1.23 | CGU(R) | 11 | 0.81 |
| CUC(L) | 7 | 0.08 | CCC(P) | 31 | 0.89 | CAC(H) | 29 | 0.77 | CGC(R) | 2 | 0.15 |
| CUA(L) | 76 | 0.85 | CCA(P) | 21 | 0.6 | CAA(Q) | 40 | 1.57 | CGA(R) | 37 | 2.74 |
| CUG(L) | 10 | 0.11 | CCG(P) | 2 | 0.06 | CAG(Q) | 11 | 0.43 | CGG(R) | 4 | 0.3 |
| AUU(I) | 275 | 1.67 | ACU(T) | 82 | 1.82 | AAU(N) | 108 | 1.38 | AGU(S) | 72 | 1.72 |
| AUC(I) | 55 | 0.33 | ACC(T) | 26 | 0.58 | AAC(N) | 49 | 0.62 | AGC(S) | 13 | 0.31 |
| AUA(M) | 203 | 1.53 | ACA(T) | 65 | 1.44 | AAA(K) | 61 | 1.33 | AGA(S) | 53 | 1.27 |
| AUG(M) | 62 | 0.47 | ACG(T) | 7 | 0.16 | AAG(K) | 31 | 0.67 | AGG(S) | 0 | 0 |
| GUU(V) | 106 | 1.84 | GCU(A) | 105 | 2.31 | GAU(D) | 42 | 1.24 | GGU(G) | 62 | 1.12 |
| GUC(V) | 17 | 0.3 | GCC(A) | 21 | 0.46 | GAC(D) | 26 | 0.76 | GGC(G) | 17 | 0.31 |
| GUA(V) | 78 | 1.36 | GCA(A) | 45 | 0.99 | GAA(E) | 51 | 1.16 | GGA(G) | 71 | 1.29 |
| GUG(V) | 29 | 0.5 | GCG(A) | 11 | 0.24 | GAG(E) | 37 | 0.84 | GGG(G) | 71 | 1.29 |

**Figure S1.** Predicted secondary structure of the 22 tRNAs in the *P. austroasiaticus* mitochondrial genome. Watson-Crick base pairings are indicated by dashes (-): GU pairs are indicated by solid dots: and AU, AA, and UU are indicated by hollow dots.

**Figure S2.** Predicted secondary structure of the 22 tRNAs in the *P. subterraneus* mitochondrial genome. Watson-Crick base pairings are indicated by dashes (-): GU pairs are indicated by solid dots: and AU, AA, and UU are indicated by hollow dots.

**Figure S3.** Predicted secondary structure of the 22 tRNAs in the *P. carabi* BJ1 mitochondrial genome. Watson-Crick base pairings are indicated by dashes (-): GU pairs are indicated by solid dots: and AU, AA, and UU are indicated by hollow dots.

**Figure S4.** Predicted secondary structure of the 22 tRNAs in the *P. carabi* BJ2 mitochondrial genome. Watson-Crick base pairings are indicated by dashes (-): GU pairs are indicated by solid dots: and AU, AA, and UU are indicated by hollow dots.

**Figure S5.** Maximum Likelihood (ML) phylogenetic tree inferred from 13 mitochondrial protein-coding genes and two rRNA genes.

**Figure S6.** Bayesian Inference (BI) phylogenetic tree inferred from 13 mitochondrial protein-coding genes and two rRNA genes.
